# Mental health-related telemedicine interventions for pregnant women and new mothers: a systematic literature review

**DOI:** 10.1186/s12888-023-04790-0

**Published:** 2023-04-28

**Authors:** Ulrike Stentzel, Hans J. Grabe, Silke Schmidt, Samuel Tomczyk, Neeltje van den Berg, Angelika Beyer

**Affiliations:** 1 Institute for Community Medicine, University Medicine Greifswald, Section Epidemiology of Health Care and Community Health, Ellernholzstraße 1-2, 17489 Greifswald, Germany; 2 Department of Psychiatry and Psychotherapy, University Medicine Greifswald, Ellernholzstraße 1-2, 17489 Greifswald, Germany; 3 Department Health and Prevention, Institute of Psychology, University of Greifswald, Greifswald, Germany

**Keywords:** “Telemedicine, “Telepsychiatry, “eHealth, “Pregnant women, “Pregnancy, “Postpartum period, “Psychiatry, “Mental health, “Mental strain, “Mental stress, “Mental problems

## Abstract

**Background:**

Pregnancy and the postpartum period are times when women are at increased risk for depression and mental problems. This may also negatively affect the foetus. Thus, there is a need for interventions with low-threshold access and care. Telemedicine interventions are a promising approach to address these issues. This systematic literature review examined the efficacy of telemedicine interventions for pregnant women and/or new mothers to address mental health-related outcomes. The primary objective was to analyse whether telemedicine interventions can reduce mental health problems in pregnant women and new mothers. The secondary aim was to clarify the impact of type of interventions, their frequency and their targets.

**Methods:**

Inclusion criteria: randomized controlled trials, with participants being pregnant women and/or new mothers (with infants up to twelve months), involving telemedicine interventions of any kind (e.g. websites, apps, chats, telephone), and addressing any mental health-related outcomes like depression, postnatal depression, anxiety, stress and others. Search terms were pregnant women, new mothers, telemedicine, RCT (randomised controlled trials), mental stress as well as numerous synonyms including medical subject headings. The literature search was conducted within the databases PubMed, Cochrane Library, Web of Science and PsycINFO. Screening, inclusion of records and data extraction were performed by two researchers according to the PRISMA guidelines, using the online tool CADIMA.

**Results:**

Forty four articles were included. A majority (62%) reported significantly improved mental health-related outcomes for participants receiving telemedicine interventions compared to control. In particular (internet-delivered) Cognitive Behavioural Therapy was successful for depression and stress, and peer support improved outcomes for postnatal depression and anxiety. Interventions with preventive approaches and interventions aimed at symptom reduction were largely successful. For the most part there was no significant improvement in the symptoms of anxiety.

**Conclusion:**

Telemedicine interventions evaluated within RCTs were mostly successful. However, they need to be designed to specifically target a certain mental health issue because there is no one-size-fits-all approach. Further research should focus on which specific interventions are appropriate for which mental health outcomes in terms of intervention delivery modes, content, target approaches, etc. Further investigation is needed, in particular with regard to anxiety.

**Supplementary Information:**

The online version contains supplementary material available at 10.1186/s12888-023-04790-0.

## Background

Pregnancy and the postpartum period are times when women are at greater risk of suffering from depression and other mental health problems. Due to the fact that hormonal changes increase the risk for mental disorders, women are more prone to affective disorders during their childbearing years [1]. In high-income countries, 10% of pregnant women and 13% of new mothers suffer from some type of mental disorders [2]. For example, in Germany, the analysis of reimbursement data of a German statutory health insurance showed a prevalence of 9.3% for depression, 16.9% for anxiety disorder, 24.2% for a somatoform/dissociative disorder, and 11.7% for acute stress reactions in pregnant women. In total, 43.6% of 38,174 pregnant women had at least one ailment in the field of mental disorders in 2008 [3]. Within the course of the pregnancy, the risk for depression was higher in the second (12.8%) and third trimester (12.0%) than in the first (7.4%) [4]. The incidence and prevalence of postpartum depression among healthy mothers without prior history of depression was ascertained through a review by Shorey et al. The incidence was 12% [95% CI 0.04–0.20] and the overall prevalence was 17% [95% CI 0.15–0.20] [5]. It is known, that the prevalence is higher in low-to middle-income countries than in high-income countries [2, 6].

An untreated mental disorder during pregnancy can affect not just the mother but also the foetus [7] and it also has implications for the postpartum development [8]. Untreated mental disorders can lead to and are connected to the following: poor nutrition and impaired self-care, a failure to follow medical and prenatal guidelines, a worsening of comorbid somatic illness, pre-eclampsia, an increased exposure to tobacco, alcohol and drugs, postpartum psychiatric complications, and they can also affect family members, for instance, the quality of relationships [6, 8–11]. Consequences of maternal mental disorders can affect the foetus, leading to preterm birth, lower birth weight, reduced foetal growth, a higher risk for spontaneous abortion, and a higher risk for operative or instrumental delivery [8, 11–13]. The result of this is that newborns have a higher risk for poor neonatal adaptation, and stays at neonatal intermediate care units are more likely. Apgar scores can be lower and head circumferences can be smaller. They more often show growth retardation, a slowed mental development, excessive crying, irritability, hostility and erratic sleep [8]. Furthermore, untreated mental disorders in the mothers can affect the mother–child-bonding [14]. The study of Ohoka et al. showed that the mother-to-infant-bonding is less pronounced in women who suffer from depression during pregnancy, furthermore, that bonding decreased after delivery, and then went up at 1 month postpartum [15]. The negative effects on children's development caused by low mother–child bonding are well known [16]. It can cause cognitive delays, behavioural and emotional difficulties, maladaptive social interactions and difficulties with affect regulation. It is also known that children with mentally ill parents have higher risks of developing a psychopathology of their own [7, 17–19]. Affected children display more fear and anxiety, higher rates of Attention Deficit Hyperactivity Disorder, higher impulsivity and lower intelligence quotient at age 14—15 years [8]. Hence, having mental disorders during pregnancy, puerperium and postpartum is serious both for the mother and for the child [7]. Diagnostics and treatment are needed as early as possible [11, 20, 21]. Psychological and psychosocial interventions for postnatal depression were found to be both effective and cost-effective [6]. It was also found that robust effects were achieved using CBT or behavioural activation for perinatal depression [6]. However, treatment rates were low [22] both during pregnancy as well as postpartum [23]. Flynn et al. found, that 65% of pregnant women with a current major depressive disorder were not receiving any depression treatment [24]. Stigma, risks of using antidepressant medications during pregnancy, lack of time, childcare issues, as well as limited resources, particularly in rural regions, high amounts of stress, lack of transportation and inadequate support were identified as impeding barriers to care [6, 21, 25].

To achieve conditions conducive to the healthy development of children, there is a need for interventions with low threshold access and care in order to reach affected pregnant women and new mothers. To address the issues mentioned above, the use of telemedicine is a promising approach. Telemedicine interventions can replace face-to-face care but can also support and reduce the burden on regular health care providers. An added bonus of telemedicine is the relative ease of access for rural and remote residents. To our knowledge, there are currently few systematic reviews and meta-analyses in the literature that have dealt specifically with the topic of mental health and telemedicine interventions for pregnant women or new mothers [26–31]. Existing reviews focused on specific intervention contents like internet-delivered Cognitive Behavioural Therapy (iCBT) [27, 31] on specific diagnoses like depression and anxiety [27, 28, 30, 31] or on specific modalities like internet-delivered interventions [26, 29]. Moreover, few studies have focused on mental health during pregnancy.

This article aimed to review studies that have undertaken any telemedical interventions addressing the mental health of pregnant women and/or women in the postpartum stage with infants. A review protocol was written in advance and registered in the systematic verification protocol database (PROSPERO registry no.166180). The final version of the protocol with all the amendments made is provided as supplementary file S1.

The details of successful telemedicine interventions were be explored by addressing the following specific review questions:

- Primary research question: Can telemedicine interventions reduce mental health problems in pregnant women and new mothers?
- Secondary research questions:
  1. Which kinds of telemedicine interventions (applications, modalities, targets) were developed and conducted for the specified patient group?
  2. For which kinds of mental problems, mental disorders, mental diseases or mental diagnoses in pregnant women and new mothers were the telemedicine interventions developed and evaluated?
  3. Which delivery modes were successful (e.g., intervention provided via phone calls, apps, web portals)? Were there differences between interventions using only a technical device and interventions with (additional) personal support? Were there differences in success with regard to the pre- or postpartum delivery timeframe?
  4. How was the participants’ acceptance of the intervention?
  5. Were implementation barriers reported and if so, what kind?
  6. Which kinds of mental health-related outcomes were addressed with telemedicine interventions?

## Main text

### Methods

The review was performed according to the PRISMA guidelines [32].

#### Eligibility criteria

This review considered randomized controlled trials (RCT) that involved pregnant women or new mothers (with infants up to twelve months) that were affected by mental problems or some kind of mental disorder/disease. All kinds of mental health-related outcomes (e.g. medical outcomes, patient-oriented outcomes) were included. The interventions could be of any kind but had to be telemedicine interventions. We defined telemedicine interventions as interventions that are rendered over spatial distances via information and communication technologies, e.g. telephone calls, chats, videoconferences or interactive support systems e.g. web portals or apps for smartphones or tablets. There were no restrictions in terms of the control group (e.g., wait list, treatment as usual, …).

There were no restrictions based on the type of setting. Articles in English and German were considered. Abstracts had to be available. Because of the technological developments of telemedicine systems and devices as well as the increase of the number of households with access to the internet, the review was restricted to studies from 2007 until November 2020. This restriction of the time period reduced heterogeneity due to technical development and communication infrastructure, thus improving comparability between the studies.

The mentioned criteria resulted in a PICO framework that contained numerous synonyms including medical subject headings. This process was then followed for the search as well as for screening and article inclusion: P(opulation) = “pregnant women” and “new mothers”, I(ntervention) = “telemedicine”, C(omparator) = “RCT”, O(utcome) = “mental stress”.

#### Databases and search

The literature search was conducted within the databases PubMed, Cochrane Library, Web of Science and PsycINFO. The search term contained the terms “telemedicine”, “mental stress”, “pregnant women” and “new mothers” and numerous synonyms for these terms (see Table 1), including medical subject headings (MeSH). Two searches were performed in each database, one with all terms for telemedicine and mental health problems for pregnant women, and one for new mothers. The exact terms for each database are provided in supplementary file S2.

**Table 1:** Search terms

Concept	Search terms
Telemedicine	telemedicine, telemedical, tele care, Mobile Health, telehealth, telepsychiatry, eHealth, mhealth, phone call, text messages, smartphone, tablet, apps, web apps, web portal, internet
mental stress	mentally stressed, mental health, mental disorder, mental disease, mentally ill, psychological, psychological impairment, psychologically affected, psychologically stressed, psychological disorder, psychological distress, psychiatric disorder, psychiatric disease, depression, anxiety, panic disorder
pregnant women	pregnancy, pregnant, prenatal, prepartal, antenatal, prepartum, peripartum, gestation, gravidic, childbearing
new mothers	young mothers, new mums/moms, young mums/moms, peripartum, postnatal, postpartal, postpartum, puerperium

#### Screening, extraction and critical appraisal

The review was conducted using CADIMA, an open access online tool that supports the conducting and reporting of systematic reviews and systematic maps [33]. CADIMA facilitates the.

- set up of the review (including to define the research question)
- protocol development (including inclusion and exclusion criteria)
- duplicate checking
- article screening/article selection (for a team of reviewers)
- and critical appraisal/risk of bias assessments.

The literature search results from the databases were uploaded in CADIMA. The screening for the inclusion criteria was independently conducted within CADIMA by two reviewers (the first and last author) following the PICO framework. Both researchers performed several rounds of consistency training. The result was a Kappa value of 0.7 indicating a ‘good’ consistency. Differences in the screening results were discussed and solved in researcher team meetings. The full texts of the articles that met the inclusion criteria based on their titles and abstracts as well as those that were inconclusive were obtained by US. For each of the articles included, a thorough extraction of relevant information was conducted by AB and US using a structured data extraction worksheet. To assess the possible risk of bias, the revised Cochrane risk-of-bias tool for randomized trials (RoB 2 https://methods.cochrane.org/bias/resources/rob-2-revised-cochrane-risk-bias-tool-randomized-trials) was used [34]. The authors AB and US performed several rounds of both data extraction and assessments of risk of bias together in order to achieve consistency in the procedures.

#### Evaluation

Several studies examined more than one mental health-related outcome, the results of which may or may not be significant. In order to compare the different characteristics of the studies with the success of the telemedicine interventions, the characteristic ‘being successful’ had to be defined. It was operationalized through a variable ‘any significant result’ in which the results were combined and categorized into ‘articles with significantly improved scores compared to control’, ‘articles without significant effect between groups’ and ‘articles with significantly higher (i.e. worse) scores compared to control’ regarding in-between group differences (telemedicine intervention versus treatment as usual (TAU) or waitlist control or another intervention). Differences between pre- and post-treatment effects were not considered as evidence for the success of the telemedicine intervention because several studies showed that the participants in control groups also improved over time [20, 35–41], especially with regard to postnatal depression.

The evaluation, the presentation of the results and the discussion involved several steps: Step 1 was a general description of all the articles included. In step 2, the articles that reported significantly improved results were examined further with regard to the secondary objectives from question two and six. The focus was on what mental health-related outcomes had been found and which of them could be addressed with telemedicine (multiple entries per article were possible). In step 3, the focus was on the secondary objectives from question one and three, which addressed characteristics of the interventions like the intervention timeframe (‘study-inclusion’, ‘administration’, ‘prevention/treatment’), kind of intervention (‘monitoring’, ‘self-help tool’, ‘support’, ‘treatment’), target of intervention (‘managing and coping with stress’, ‘preventing stress/mental health impairment’, ‘strengthening mental health’, ‘symptom reduction’, ‘the main intervention target not in the field of mental health’), content of intervention (i. e. ‘iCBT’, ‘education’, ‘peer support’, etc.), delivery mode (‘app’, ‘emails’, ‘internet website’, ‘telephone’, ‘videoconference’) and different modes of contacts (i.e. ‘personal contact: yes/no’ or ‘kind of contact’). In this step it was possible for articles to address one, two or more, or none of the characteristics. In step 4, the four most frequently reported mental health-related outcomes from step 2 were linked to the characteristics from step 3. In step 5, the focus was on articles that mentioned feasibility, acceptance and implementation barriers (see secondary objectives, questions four and five).

## Results

**Fig. 1:**
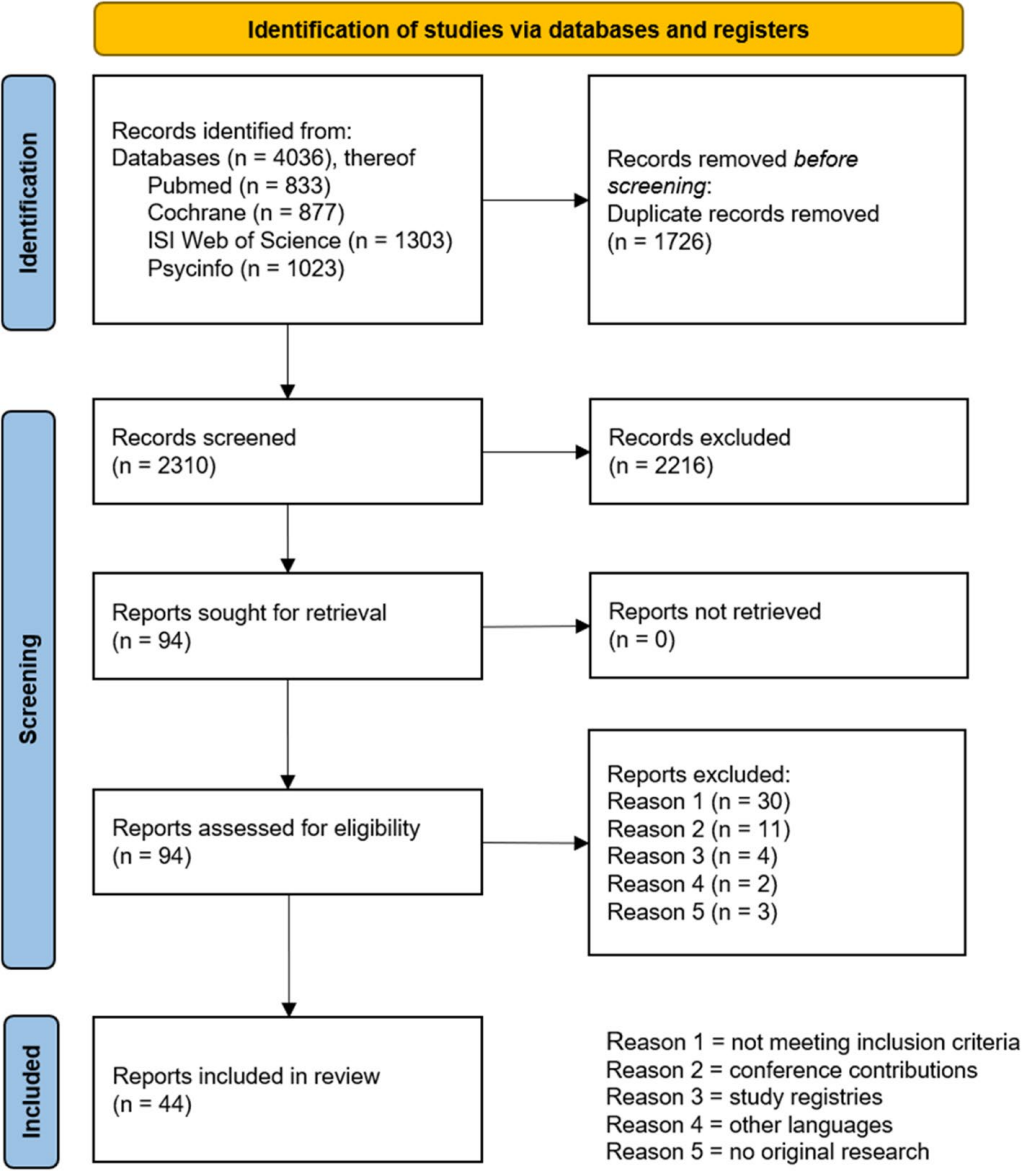
Flow diagram according to PRISMA 2020 guidelines

The searches in all the databases identified 4036 records in total. Figure 1 presents the flow diagram according to the PRISMA 2020 guidelines. After removing duplicate and ineligible records, the remaining 44 reports (solely articles) were included in this review (supplementary file S3).

### Step 1: General results

Eligible RCTs came from all over the world. Sorted by continent in decreasing order most studies were conducted in North America (36%, *n* = 16 [35, 41–55]), Europe (30%, *n* = 12 [20, 37, 40, 56–64]), Asia (14%, *n* = 6 [36, 65–69]), Australia (9%, *n* = 4 [39, 70–72]). One article originated in Africa [73] and several studies (*n* = 4, [74–77]) were conducted on two different continents. The articles were published between 2009 and 2020, whereby 2019 was by far the most frequent publication year with 13 articles. Women were recruited in six different settings: 45% (*n* = 20, [20, 35, 38–40, 43, 48, 49, 52, 54, 57–63, 70, 71, 76]) using analogue or digital advertisements and invitations, 5% (*n* = 2, [56, 64]) in antenatal childbirth classes, 2% (*n* = 1, [41]) in Head Start classrooms (classes for early childhood education), 18% (*n* = 8, [42, 44, 53, 65, 66, 68, 75, 77]) during in-patient clinic stays, 25% (*n* = 11, [36, 37, 45–47, 50, 51, 67, 69, 72, 74]) during regular checks and 5% (*n* = 2, [55, 73]) within specialized care.

The studies’ sample size ranged from 24 to 1340 women, with a mean of 242, a standard deviation of 302, a median of 132, and an interquartile range of 68 to 247.

The control groups comprised 57% TAU (*n* = 25, [20, 35–39, 42, 45, 46, 51, 53, 56, 63, 66–75, 77]), 23% waitlist controls (*n* = 10, [41, 49, 52, 57–62, 65]), 7% information only controls (*n* = 3, [43, 50, 54]), 7% another intervention (*n* = 3, [44, 48, 76]), 5% distraction controls (*n* = 2, [40, 64]), and 2% placebo training (*n* = 1, [47]). There were three three-arm studies, two of these had two intervention arms and one TAU arm [35, 75], and one had two intervention arms and a waitlist control arm [49].

Ninety-three % of the studies (*n* = 41, [20, 35, 37–66, 69–77]) addressed pregnant women and/or new mothers, whereas in 7% (*n* = 3, [36, 67, 68]) both mothers and fathers were included.

It was found that 75% (*n* = 33 [20, 36–39, 41, 43, 45–49, 51, 52, 54, 55, 58–66, 69–74, 76, 77]) of the articles described mental health-related outcomes as primary and 25% (*n* = 11 [35, 40, 42, 44, 50, 53, 56, 57, 67, 68, 75]) as a secondary outcome. In the following, the results are not described separately for primary and secondary outcomes.

### Step 2: Mental health-related outcomes addressed

The 44 articles included in this literature review addressed the following mental health-related outcomes: positive mental health, post-traumatic stress disorder, depression, postnatal depression, anxiety, quality of life, wellbeing, stress, emotion regulation / psychological flexibility and mindfulness / self-compassion (total count *n* = 106). Supplementary file S4 provides the measuring instruments that were used in the articles to operationalize the respective mental health-related outcome. For 52 of the 106 mental health-related outcomes significantly improved scores for the intervention group compared to the control or waitlist group were found (Table 2). For 53 mental health-related outcomes no significant effects were reported. One article only evaluated the intervention results in a descriptive form [48]. Another article did not report the mental health-related outcome results [65]. Hence, in the following presentation of results, those two articles were categorised as having “no significant effects”. One article reported significantly higher (i.e. worse) scores for postnatal depression [72]. Of all the included articles *n* = 44, *n* = 29 (66%) reported results that reached a level of significance. Thereof *n* = 28 (64%) [20, 36, 37, 39, 41, 45, 46, 51, 52, 54, 56–63, 65–67, 69–71, 73–75, 77] reported significantly improved scores compared to the control. Table 2 shows the assessment of the overall risk of bias (low, some concerns, high, no information available) of the respective publications. Further results regarding the risk of bias assessment are provided in the supplementary file S5.

**Table 2:** Overall number of reported mental health-related outcomes and the articles’ risk of bias

Mental health-related outcomes	Articles with significantly improved scores compared to control					Articles without a significant difference in effect between groups				Articles with significantly higher (i.e. worse) scores compared to control	
	N	[Literature References]				N	[Literature References]			N	[Literature References]
		RoB: low	RoB: con-cerns	RoB: high	RoB: n.i		RoB: low	RoB: con-cerns	RoB: high		RoB: low
Postnatal depression	14	[20, 46, 52, 58, 66, 67, 75, 77]	[36]	[37, 39, 60, 63, 65]		13	[40, 42–44, 50, 53, 54, 68, 70]	[55, 61]	[38, 51]	1	[72]
Anxiety	8	[20, 46, 52, 58, 67, 69, 77]		[39]		16 ^a, b^	[40, 47, 59, 62, 70, 71, 75]	[36, 55, 61, 64]	[38, 48, 60, 65, 76]		
Depression	11	[20, 41, 52, 54, 62, 69, 71, 77]	[36]	[39, 51]		10 ^a, b^	[35, 43, 47, 59, 70]		[38, 48, 49, 65, 76]		
Stress	5	[45, 52, 70, 71]		[39]		7	[40, 44, 72]	[36, 55, 64]	[60]		
Mindfulness AND/OR self-compassion	4	[59, 69]	[57]	[65]		1			[76]		
Wellbeing	4	[59, 74]		[65]	[56]						
Emotion regulation AND/OR psychological flexibility	2	[73]	[57]								
Quality of life	2	[52]		[39]		5	[20, 35, 62, 70]	[36],			
Positive mental health	1		[61]			1			[51]		
Post-traumatic stress disorder	1	[62]									
Total	52					53					1

*RoB* Overall assessment of risk of bias (low, some concerns, high), *n.i.* No information available

^a^one article reported descriptive statistics: reduction in symptoms (no significance calculated)[48]

^b^one article did not report outcome results[65]

### Step 3: Characteristics of telemedicine interventions

Twenty-four articles reported about studies where recruitment occurred during pregnancy. The timeframe for study inclusion varied between the first trimester of gestation and the entire pregnancy. For 20 articles, inclusion in the respective study was only possible at the time when the women gave birth or in the months thereafter. For this, the possible time-frame varied between recruitment taking place at postpartum wards shortly before or after the women had given birth any time until the child was 12 months old, which was the maximum age for inclusion. Table 3 shows all the articles that reported at least one outcome with significant improvement compared to the control group.

**Table 3:** Number of articles with significant improvement of mental health-related outcomes according to the timeframe for inclusion and intervention

	Articles (n (%)) with at least one outcome with significant improvement [Literature References]	
Timeframe for inclusion in the study		
Pregnancy (*n* = 24)	13 (54.1)	[20, 36, 37, 39, 45, 56, 60, 65, 67, 69, 73, 74, 77]
Postpartum period (*n* = 20)	15 (75.0)	[41, 46, 51, 52, 54, 57–59, 61–63, 66, 70, 71, 75]
Timeframe for intervention		
Pregnancy (*n* = 14)	9 (64.3)	[20, 36, 39, 45, 56, 60, 65, 69, 73]
Postpartum period (*n* = 20)	15 (75.0)	[39, 41, 46, 51, 52, 54, 57–59, 61–63, 66, 71, 75]
Both pregnancy and postpartum (*n* = 10)	4 (40.0)	[37, 67, 74, 77]

The interventions covered in the articles were intended to either be preventive or as treatment, whereby *n* = 27 articles covered interventions with the goal of prevention and *n* = 17 articles covered treatments. Table 4 shows the findings where the intervention group had significantly better results than the control group.

**Table 4:** Articles with significant improvement of mental health-related outcomes in terms of prevention/treatment

Prevention/Treatment	Articles (n (%)) with at least one outcome with significant improvement [Literature References]	
Prevention (*n* = 27)	17 (63%)	[36, 37, 45, 46, 56–61, 65, 67, 69, 73–75, 77]
Treatment (*n* = 17)	12 (71%)	[20, 39, 41, 51, 52, 54, 62, 63, 66, 70–72]

The 44 articles were categorized according to our interpretation of the kind of intervention used [‘monitoring’ (*n* = 1 [42]), ‘self-help tool’ (*n* = 32 [20, 35–41, 43, 44, 47, 48, 50, 52, 56–65, 67, 68, 70–72, 74–76]), ‘support’ (*n* = 7 [46, 49, 53, 54, 69, 73, 77]), ‘support and monitoring’ (*n* = 1 [45]), ‘treatment’ *n* = 3 [51, 55, 66])]. Table 5 shows the articles that reported significantly improved results sorted according to the kind of intervention employed.

**Table 5:** Articles with significant improvement of mental health-related outcomes according to kind of intervention

Kind of intervention	Articles (n (%)) with at least one outcome with significant improvement [Literature References]	
Self-help tool (*n* = 32)	20 (66%)	[20, 36, 37, 39, 41, 52, 56–63, 65, 67, 70, 71, 74, 75]
Support (*n* = 7)	5 (71%)	[46, 54, 69, 73, 77]
Support and monitoring (*n* = 1)	1 (100%)	[45]
Treatment (*n* = 3)	2 (67%)	[51, 66]

Another categorization concerned the goals of the intervention: ‘managing and coping with stress’ (*n* = 3 [40, 48, 60]), ‘preventing stress/mental health impairment’ (*n* = 11 [36, 37, 43, 45, 46, 54, 57, 58, 61, 65, 77]), ‘strengthening mental health’ (*n* = 6 [56, 59, 73–76]) and ‘symptom reduction’ (*n* = 17 [20, 38, 39, 41, 47, 49, 51, 52, 55, 62–64, 66, 69–72]). In *n* = 7 articles the ‘main intervention target was not in the field of mental health [35, 42, 44, 50, 53, 67, 68]). These interventions focused on breastfeeding [42], weight reduction/control [35, 44], insomnia/sleep [50, 53], and parental self-efficacy [67, 68]. Table 6 shows the articles that reported significantly improved results.

**Table 6:** Articles with significant improvement of mental health-related outcomes according to intervention target

Intervention target	Articles (n (%)) with at least one outcome with significant improvement [Literature References]	
Managing and coping with stress (*n* = 3)	1 (33%)	[60]
Preventing stress/mental health impairment (*n* = 11)	10 (91%)	[36, 37, 45, 46, 54, 57, 58, 61, 65, 77]
Strengthening mental health (*n* = 6)	5 (83%)	[56, 59, 73–75]
Symptom reduction (*n* = 17)	11 (65%)	[20, 39, 41, 51, 52, 62, 63, 66, 69–71]
Main intervention target not in the field of mental health (*n* = 7)	1 (14%)	[67]

The 44 articles included in this review were also categorized according to the content of the intervention. The most common were ‘(internet-delivered) Cognitive Behavioural Therapy [iCBT]’ (*n* = 14 [20, 39, 41, 48, 50, 52, 54, 57, 58, 61, 62, 66, 70, 76]), ‘education lessons’ (*n* = 10 [35–37, 40, 43, 53, 67, 68, 71, 75]), ‘mindfulness’ (*n* = 5 [59, 60, 65, 69, 74]) and ‘peer support’ (*n* = 3 [46, 49, 77]). Other intervention contents were ‘Acceptance and Commitment Therapy (ACT)’ [73], ‘gaming’ [47], ‘internet-based Behavioural Activation (iBA)’ [63], relaxation exercises/mood tracking’ [56], ‘monitoring’ [42], ‘prenatal care’ [45], ‘problem solving treatment’ [38], ‘stress management’ [64], and a ‘supplemental nutrition program’ [44]. ‘Online group and education’ [72] were also mentioned only once as intervention content. Two interventions were referred to as treatment sessions without further specification (‘treatment sessions via telephone by certified nurse-midwives’, ‘videoconferencing’) [51, 55]. Table 7 shows the articles that reported significantly improved results.

**Table 7:** Articles with significant improvement of mental health-related outcomes according to intervention contents

Intervention contents	Articles (n (%)) with at least one outcome with significant improvement [Literature References]	
iCBT (*n* = 14)	11 (79%)	[20, 39, 41, 52, 54, 57, 58, 61, 62, 66, 70]
Education lessons (*n* = 10)	5 (50%)	[36, 37, 67, 71, 75]
Mindfulness (*n* = 5)	5 (100%)	[59, 60, 65, 69, 74]
Peer support (*n* = 3)	2 (67%)	[46, 77]
Acceptance and commitment therapy (ACT) (*n* = 1)	1 (100%)	[73]
Internet-based behavioural activation (iBA) (*n* = 1)	1 (100%)	[63]
Prenatal care (*n* = 1)	1 (100%)	[45]
Relaxation exercises/mood tracking (*n* = 1)	1 (100%)	[56]
Treatment sessions via telephone by certified nurse-midwives (*n* = 1)	1 (100%)	[51]

The delivery modes of the intervention as indicated in the 44 articles were as follows: ‘apps’ (*n* = 7 [35, 36, 47, 56, 68, 72, 73]), ‘emails’ (*n* = 1 [76]), ‘internet websites’ (*n* = 26 [20, 37–44, 48, 50, 52, 57–65, 69–71, 74, 75]), ‘telephone’ (*n* = 6 [46, 49, 51, 53, 54, 66]), ‘telephone and app’ (*n* = 1 [67]), ‘telephone and online community’ (*n* = 1 [45]), ‘telephone, emails or other messengers’ (*n* = 1 [77]) and ‘video conference’ (*n* = 1 [55])]. Table 8 shows the articles that reported significantly improved results.

**Table 8:** Articles with significant improvement of mental health-related outcomes according to delivery mode

Intervention delivery mode	Articles (n (%)) with at least one outcome with significant improvement [Literature References]	
App (*n* = 7)	3 (57%)	[36, 56, 73]
Emails (*n* = 1)	0	
Internet intervention: website (*n* = 26)	18 (69%)	[20, 37, 39, 41, 52, 57–63, 65, 69–71, 74, 75]
Telephone (*n* = 6)	4 (67%)	[46, 51, 54, 66]
Telephone and app (*n* = 1)	1 (100%)	[67]
Telephone and/or online community (*n* = 1)	1 (100%)	[45]
Telephone, emails or other messengers (*n* = 1)	1 (100%)	[77]
Videoconference (*n* = 1)	0	

One special point of interest was whether or not the telemedicine interventions involved any kind of personal contact. ‘Personal contacts’ were understood as a communication interaction between the participants and study personnel or peers in this review. The intervention included such a personal contact in 29 of the articles [20, 35, 36, 38, 40–42, 44–46, 48–55, 62–64, 66–69, 71, 72, 75, 77]. The remaining 15 articles did not [37, 39, 43, 47, 56–61, 65, 70, 73, 74, 76]. Table 9 shows the articles that reported significantly improved results.

**Table 9:** Articles with significant improvement of mental health-related outcomes according to personal contacts

Personal contacts – yes/no	Articles (n (%)) with at least one outcome with significant improvement [Literature References]	
Yes (*n* = 29)	16 (55%)	[20, 36, 41, 45, 46, 51, 52, 54, 62, 63, 66, 67, 69, 71, 75, 77]
No (*n* = 15)	12 (80%)	[37, 39, 56–61, 65, 70, 73, 74]

Contacts restricted to ‘purely technical support’ reported *n* = 9 articles [37, 39, 44, 57, 58, 61, 70, 74, 76], thereof *n* = 1 ‘automated feedback’ [44], *n* = 2 ‘delivery by email’ [37, 74], *n* = 5 ‘reminders or reminders plus program-related support’ [39, 57, 58, 61, 76], and *n* = 1 ‘technical assistance’ [70].

For the above-mentioned articles with personal contacts (*n* = 29), our categorization was based on (a) contact options, (b) the profession of the person involved in the contact and (c) the form the contact was offered in.

(a) Some studies offered several contact options, which we identified as different contact options: ‘private texts or messages’ (*n* = 8 [20, 35, 36, 44, 64, 72, 75, 77]), ‘feedbacks or reviews’ (*n* = 8 [20, 38, 40, 48, 50, 52, 62, 64]), ‘chat rooms, forums, posts, comments, likes’ (*n* = 7 [40, 44, 48, 63, 68, 71, 75]), ‘emails’ (*n* = 6 [35, 38, 40, 45, 71, 77]), ‘answers to queries or questions’ (*n* = 2 [63, 67]), ‘support or encouragement’ (*n* = 3 [20, 52, 62]), 2 studies provided ‘videoconferences’ [55, 69], one ‘reminder calls’ [75] and one study provided ‘guidance’ [50].

(b) Contact was made in the individual studies by people in different professions (multiple professions were possible for one article): most often by ‘peers’ (*n* = 8 [40, 46, 48, 49, 68, 71, 75, 77]), followed by ‘therapists/psychologists’(*n* = 6 [20, 40, 52, 55, 62, 64]), then ‘interventionists/research assistants or interventionists and peers’ (*n* = 6 [35, 42, 44, 50, 63, 75]), and *n* = 4 for the three following professions: by a ‘coach’ [38, 41, 54, 71], by a ‘midwife’ [51, 66–68] and by a ‘nurse’ [45, 53, 69, 72]. One article described the person who made the contact as an ‘obstetrician’[36].

(c) Personal contacts were provided in the form of ‘bilateral contacts’ (*n* = 20 [20, 35, 36, 38, 41, 42, 45, 46, 49–55, 62, 64, 66, 69, 77]), as a ‘forum’, where participants could post, read, and comment on messages, share experiences and exchange views and information with other participants and sometimes also with study staff (*n* = 3 [48, 67, 72]), as both ‘contact and forum’ (*n* = 4 [40, 68, 71, 75]), once as ‘contact, forum and group meeting’ [44] and once as a ‘forum with idiographic answers to queries’ [63]. Table 10 shows the findings with significantly better results according to the kind of personal contact.

**Table 10:** Articles with significant improvement of mental health-related outcomes according to kind of personal contact

Kind of personal contact	Articles (n (%)) with at least one outcome with significant improvement [Literature References]	
Bilateral contacts (*n* = 20)	12 (60%)	[20, 36, 41, 45, 46, 51, 52, 54, 62, 66, 69, 77]
Forum (*n* = 3)	2 (67%)	[67, 72]
Contact and forum (*n* = 4)	2 (50%)	[71, 75]
Forum with idiographic answers to queries (*n* = 1)	1 (100%)	[63]

### Step 4: Results for the most common mental health-related outcomes

Some mental health-related outcomes were more common than others. Common outcomes were depression, postnatal depression, anxiety and stress and thus these outcomes were examined further. More specifically, the results concerning these four outcomes were examined in relation to the characteristics ‘kind of intervention’, ‘intervention target’, ‘intervention content’, ‘delivery mode’ and ‘personal contact’.

#### Depression

Nineteen articles reported levels of depression – operationalized with different measuring instruments for depression, see supplementary file S4 – as a mental health-related outcome [20, 35, 36, 38, 39, 41, 43, 47, 49, 51, 52, 54, 59, 62, 69–71, 76, 77]. Eleven articles reported at least one outcome with a significant improvement. Of those, 73% showed an overall low risk of bias, 9% some concerns and 18% had an overall high risk of bias (see also Table 2). Table 11 breaks down the findings with significantly better results for depression according to the characteristics mentioned above. The target of ‘strengthening mental health’ was also considered, but no article reported significant results in this regard (*n* = 2 [59, 76]). One article’s intervention target was categorized as ‘main intervention target not in the field of mental health’ (*n* = 1 [35]) and, furthermore, showed no significant results. One article mentioned email as the delivery mode but showed no significant results [76].

**Table 11:** Articles reporting significant improvement of depression after intervention

Depression (*n* = 19 articles in total)	Articles (n (%)) with at least one outcome with significant improvement [Literature References]	
**Kind of intervention**		
Self-help tool (*n* = 13)	7 (54%)	[20, 36, 39, 41, 52, 62, 71]
Support (*n* = 4)	3 (75%)	[54, 69, 77]
Treatment (*n* = 1)	1 (100%)	[51]
**Intervention target**		
Preventing stress/mental health impairment (*n* = 4)	3 (75%)	[36, 54, 77]
Symptom reduction (*n* = 12)	8 (67%)	[20, 39, 41, 51, 52, 62, 69, 71]
**Intervention contents**		
iCBT (*n* = 8)	6 (75%)	[20, 39, 41, 52, 54, 62]
Education lessons (*n* = 4)	2 (50%)	[36, 71]
Mindfulness (*n* = 2)	1 (50%)	[69]
Peer support (*n* = 2)	1 (50%)	[77]
Treatment sessions via telephone by certified nurse-midwives (*n* = 1)	1 (100%)	[51]
**Delivery mode**		
App (*n* = 3)	1 (33%)	[36]
Internet intervention: website (*n* = 11)	7 (64%)	[20, 39, 41, 52, 62, 69, 71]
Telephone (*n* = 3)	2 (67%)	[51, 54]
Telephone, emails or other messengers (*n* = 1)	1 (100%)	[77]
**Personal contacts**		
Yes (*n* = 13)	10 (77%)	[20, 36, 41, 51, 52, 54, 62, 69, 71, 77]
No (*n* = 6)	1 (17%)	[39]

#### Postnatal depression

Twenty-eight articles reported levels of postnatal depression as a mental health-related outcome [20, 36–40, 42–44, 46, 50–55, 58, 60, 61, 63, 65–68, 70, 72, 75, 77]. Fourteen articles reported at least one outcome with significant improvement. Of those, 57% showed an overall low risk of bias, 7% some concerns and 36% had an overall high risk of bias (see also Table 2). The measuring instruments used for postnatal depression are shown in the supplementary file S4. One article reported significant worse scores for the intervention group [72]. This intervention was a ‘self-help tool’ and targeted ‘symptom reduction’, the delivery mode was an ‘app’ and the intervention content was ‘online group meetings and education’ and included personal contacts. One article named ‘monitoring’ as the kind of intervention used but it showed no significant results [42]. Another used ‘videoconference’ as its delivery mode, but reported no significant effects [55]. Table 12 shows the findings with significantly better results for postnatal depression.

**Table 12:** Articles reporting significant improvement of postnatal depression after intervention

Postnatal depression (*n* = 28 articles in total)	Articles (n (%)) with at least one outcome with significant improvement [Literature References]	
**Kind of intervention**		
Self-help tool (*n* = 20)	11 (55%)	[20, 36, 37, 39, 52, 58, 60, 63, 65, 67, 75]
Support (*n* = 4)	2 (50%)	[46, 77]
Treatment (*n* = 3)	1 (33%)	[66]
**Intervention target**		
Managing and coping with stress (*n* = 2)	1 (50%)	[60]
Preventing stress/mental health impairment (*n* = 9)	6 (67%)	[36, 37, 46, 58, 65, 77]
Strengthening mental health (*n* = 1)	1 (100%)	[75]
Symptom reduction (*n* = 10)	5 (50%)	[20, 39, 52, 63, 66]
Main intervention target not in the field of mental health (*n* = 6)	1 (17%)	[67]
**Intervention contents**		
iCBT (*n* = 9)	5 (56%)	[20, 39, 52, 58, 66]
Education lessons (*n* = 8)	4 (50%)	[36, 37, 67, 75]
Internet-based behavioural activation (iBA) (*n* = 1)	1 (100%)	[63]
Mindfulness (*n* = 2)	2 (100%)	[60, 65]
Peer support (*n* = 2)	2 (100%)	[46, 77]
**Delivery mode**		
App (*n* = 3)	1 (33%)	[36]
Internet intervention: website (*n* = 17)	9 (53%)	[20, 37, 39, 52, 58, 60, 63, 65, 75]
Telephone (*n* = 5)	2 (40%)	[46, 66]
Telephone and app (*n* = 1)	1 (100%)	[67]
Telephone, emails or other messengers (*n* = 1)	1 (100%)	[77]
**Personal contacts**		
Yes (*n* = 20)	9 (45%)	[20, 36, 46, 52, 63, 66, 67, 75, 77]
No (*n* = 8)	5 (63%)	[37, 39, 58, 60, 65]

#### Anxiety

Twenty-two articles reported levels of anxiety as a mental health-related outcome [20, 36, 38–40, 46, 47, 52, 55, 58–62, 64, 67, 69–71, 75–77] (for measuring instruments see supplementary file S4). Eight articles reported at least one outcome with significant improvement. Of those, 87.5% showed an overall low risk of bias and 12.5% an overall high risk of bias (see also Table 2). One article reported ‘treatment’ as the kind of intervention, but it had no significant effects [55]. The targets of ‘managing and coping with stress’ (*n* = 2 [40, 60]) and ‘strengthening mental health’ (*n* = 3 [59, 75, 76]) were also considered but there were no articles that reported significant results. Intervention contents without significant results were ‘gaming’ (*n* = 1 [47]), ‘problem solving treatment’ (*n* = 1 [38]), ‘stress management’ (*n* = 1 [64]) and ‘treatment sessions via videoconference’ (*n* = 1 [55]). No significant effects were reported for the following modes of delivery: ‘apps’ (*n* = 2 [36, 47]), ‘email’ (*n* = 1 [76]) and ‘videoconference’ (*n* = 1 [55]). Table 13 shows the findings with significantly better results for anxiety.

**Table 13:** Articles reporting significant improvement of anxiety after intervention

Anxiety (*n* = 22 articles in total)	Articles (n (%)) with at least one outcome with significant improvement [Literature References]	
**Kind of intervention**		
Self-help tool (*n* = 18)	5 (28%)	[20, 39, 52, 58, 67]
Support (*n* = 3)	3 (100%)	[46, 69, 77]
**Intervention target**		
Preventing stress/mental health impairment (*n* = 5)	3 (60%)	[46, 58, 77]
Symptom reduction (*n* = 11)	4 (36%)	[20, 39, 52, 69]
Main intervention target was not in the field of mental health (*n* = 2)	1 (50%)	[67]
**Intervention contents**		
iCBT (*n* = 8)	4 (50%)	[20, 39, 52, 58]
Education lessons (*n* = 5)	1 (20%)	[67]
Mindfulness (*n* = 3)	1 (33%)	[69]
Peer support (*n* = 2)	2 (100%)	[46, 77]
**Delivery mode**		
Internet intervention: website (*n* = 15)	5 (33%)	[20, 39, 52, 58, 69]
Telephone (*n* = 1)	1 (100%)	[46]
Telephone and app (*n* = 1)	1 (100%)	[67]
Telephone, emails or other messengers (*n* = 1)	1 (100%)	[77]
**Personal contacts**		
Yes (*n* = 13)	6 (43%)	[20, 46, 52, 67, 69, 77]
No (*n* = 8)	2 (25%)	[39, 58]

#### Stress

Twelve articles reported levels of stress as a mental health-related outcome [36, 39, 40, 44, 45, 52, 55, 60, 64, 70–72] (for measuring instruments see the supplementary file S4). Five articles reported at least one outcome with significant improvement. Of those articles, 80% showed an overall low risk of bias and 20% had an overall high risk of bias (see also Table 2). One article with ‘treatment’ as the kind of intervention reported no significant effects [55]. Two articles named ‘managing and coping with stress’ as the target of the interventions, but no significant effects were reported [40, 60]. Another article that considered stress reported no significant results, however in this case the ‘main intervention target was not in the field of mental health’ [44]. Regarding stress, each of the following intervention contents were mentioned once, but did not show significant results: ‘mindfulness’ [60], ‘online group meeting and education’ [72], ‘stress management’ [64], ‘supplemental nutrition program’ [44] and ‘treatment session via videoconference’ [55]. One article mentioned the use of ‘videoconference’ as the delivery mode, but it showed no significant effects [55]. There were two articles which named ‘apps’ as the delivery mode, however neither of them showed significant results [36, 72]. Table 14 shows the findings with significantly better results for stress compared to the control group.

**Table 14:** Articles reporting significant improvement of stress after intervention

Stress (*n* = 12 articles in total)	Articles (n (%)) with at least one outcome with significant improvement [Literature References]	
**Kind of intervention**		
Self-help tool (*n* = 10)	4 (40%)	[39, 52, 70, 71]
Support and monitoring (*n* = 1)	1 (100%)	[45]
**Intervention target**		
Preventing stress/mental health impairment (*n* = 2)	1 (50%)	[45]
Symptom reduction (*n* = 7)	4 (57%)	[39, 52, 70, 71]
**Intervention contents**		
iCBT (*n* = 3)	3 (100%)	[39, 52, 70]
Education lessons (*n* = 3)	1 (33%)	[71]
Prenatal care (*n* = 1)	1 (100%)	[45]
**Delivery mode**		
Internet intervention: website (*n* = 8)	4 (50%)	[39, 52, 70, 71]
Telephone and/or online community (*n* = 1)	1 (100%)	[45]
**Personal contacts**		
Yes (*n* = 9)	3 (33%)	[45, 52, 71]
No (*n* = 3)	2 (67%)	[39, 70]

### Step 5: Feasibility, acceptance and implementation barriers

The feasibility of the intervention was deemed good by *n* = 16 articles [37, 39, 41, 42, 46, 49, 52, 54, 55, 59, 61, 63, 65, 68, 70, 71]. The feasibility was unclear in two articles [53, 58]. One article pointed out that the feasibility was discussed elsewhere [57]. One article reported poor feasibility [60]. However, the majority of the articles (*n* = 24) did not deal with the feasibility of the interventions [20, 35, 36, 38, 40, 43–45, 47, 48, 50, 51, 56, 62, 64, 66, 67, 69, 72–77].

The participants were mostly positive in terms of their acceptance of or satisfaction with the intervention (*n* = 23 [20, 38, 39, 41–43, 45, 46, 48, 51, 52, 54, 55, 58, 59, 61, 62, 64, 65, 68, 70–72]), however acceptance was reported as negative in two studies[49, 60], for a further two studies the acceptance was unclear [53, 63], three articles stated that information about the participants’ acceptance was reported elsewhere [37, 57, 77] and the remaining 14 studies in this review did not mention this issue [35, 36, 40, 44, 47, 50, 56, 66, 67, 69, 73–76].

Implementation barriers were not explicitly covered. However, in a total of five articles there were statements made that could be interpreted as discussion of implementation barriers because they pointed out difficulties experienced in implementing the intervention. These statements contained the following comments: ‘did not attract optimal participants’ [43], ‘difficulty contacting mothers and inability to provide in-home practical support’ [49], ‘not sufficiently attracted to Be a Mom, disliked some of the intervention features (e.g., content, design, characters)’ [58], ‘low completion rates’ [60]. One article stated that ‘telephone remains the most accessible for most people’ [46]. One article reported that the intervention was ‘easily applicable at low cost’ [38] and the other studies (*n* = 38 [20, 35–37, 39–42, 44, 45, 47, 48, 50–57, 59, 61–77]) did not report anything about this aspect.

## Discussion

This article reviews the literature on telemedicine interventions for pregnant women or new mothers in connection with mental health-related outcomes. It identified 44 RCTs that examined the impact of telemedicine interventions on a broad range of mental health outcomes, both positive (e.g., well-being, mindfulness) as well as negative (e.g., depression, anxiety).

A 62% majority of the articles included reported at least one outcome which had significantly improved compared to the control. Therefore, it seems that telemedicine interventions can reduce mental health problems in pregnant women and new mothers. This is in line with the appraisals of Nair et al. [28] and Hanach et al. [30]. However, the reviews also revealed key differences with regard to the characteristics of the intervention (e.g., timeframe, content, mode of delivery) across the different outcomes. These differences warrant more attention in order to be able to optimize telemedical interventions and find out which type of intervention works best for what outcome and when the intervention should be employed. Therefore, we will discuss the characteristics of interventions, before looking at the implications for specific mental health-related outcomes.

Caution is warranted when interpreting the results, because statistical significance is not equivalent to clinical relevance. Statistical significance without clinical relevance is not enough to assess whether patients actually benefit from an intervention [78]. There may be undetected underlying mechanisms influencing the results, e.g. changes in the structure of health care systems or guidelines.

### Characteristics of interventions

#### Timeframe – study inclusion / implementation of intervention

For the analysis of the articles a distinction was made based on the timeframe for study inclusion, namely based on whether recruitment of participants was already possible during pregnancy, or only postpartum. The timeframe for interventions varied accordingly, i.e. for the majority of the 24 articles in which women were included in the study while pregnant, the intervention was exclusively related to pregnancy (*n* = 14). For the other articles the intervention continued beyond childbirth (*n* = 10). A majority of the articles showed significant positive results for the intervention timeframes (antenatal or postpartum period), both pregnancy (54%, *n* = 13 out of 24) and postpartum period (75%, *n* = 15 out of 20). This is in line with the findings of Ashford et al. [29] and Louhgnan et al. [27] for perinatal women. Our results are also consistent with the observation of Sockol et al. [78] that CBT-interventions generally had an even greater effect in the postpartum phase than interventions in the perinatal phase. Of the articles which stated that recruitment was during pregnancy with intervention that went beyond childbirth (*n* = 10), only 40% (*n* = 4) reported significant improvement. This was surprising considering the prolonged care for the subjects. A possible explanation is that interventions focused on a particular period of time catered more specifically to the needs that prevailed at those stages. It should be noted that this finding was not in concordance with other reviews. For example, Nair et al. [28] took both perinatal and postpartum women into account in their review and found that eight out of the ten studies included reported significant improvements.

#### Timeframe—prevention/treatment

Overall, both interventions with a preventive approach as well as those treating mental health-related outcomes predominantly achieved significant effects (63 and 69% respectively). Both approaches can be considered successful. Again, Sockol et al. [78] showed that both prevention and treatment were more successful in the postpartum period or at least later in pregnancy. They suggest that this might be caused by the more immediate relevance in the postpartum period, once the infants are born. Sockol et al. [78] assumed that because the specific needs had actually arisen, the information from the therapy could now be implemented by the mothers. The burden of suffering seems to be increased postpartum. It was found that psychiatric admissions are more likely to occur in the month after birth. Munk-Olsen et al. [79] looked in the Danish Psychiatric Central Register and found an increased risk for psychiatric outpatient contacts during the first 3 months after childbirth.

#### Targets of the intervention

The majority of interventions targeted ‘preventing stress or a mental health impairment’ and ‘symptom reduction’, few had the more general goal to ‘strengthen mental health’. All three targets were mainly successful with ‘preventing stress’ ranked first, followed by ‘strengthen mental health’ and ‘symptom reduction’. However, the actual number of interventions conducted to strengthen mental health is small. These results suggest that preventing stress is a promising approach for telemedicine interventions, yet more empirical evidence is needed to support this claim.

#### Kind of intervention

The different interventions were sorted according to their type: ‘monitoring’, ‘self-help tool’, ‘support’, ‘support and monitoring’, and ‘treatment’. The vast majority were ‘self-help tools’. Overall, the ‘self-help tools’ approach had a positive impact on mental health-related outcomes, which was confirmed by significant improvements.

#### Content of the intervention

The range of intervention contents was broad: ‘iCBT’, ‘education lessons with or without mood tracking’, ‘gaming’, ‘internet-delivered compassionate mind training (iCMT)’, ‘mindfulness’, ‘self-compassion’, ‘monitoring’, ‘peer support’, ‘problem solving treatment’, ‘relaxation exercises and mood tracking’, ‘stress management’, ‘supplemental nutrition program’, ‘treatment sessions via telephone by certified nurse-midwives’. Of these interventions, several appeared in only one article. The more common intervention contents were ‘iCBT’, ‘education lessons’, ‘mindfulness’ and ‘peer support’. A majority of studies that used ‘iCBT’ and ‘mindfulness’ showed significant improvements of the outcomes. The results of ‘education lessons’ and ‘peer support’ on the other hand were inconsistent with several studies that found no significant effects and some that found significant improvements. Further investigation is needed to establish what leads to some ‘education lessons’ being successful when others are not.

#### Delivery mode

As mentioned in the methods section, we defined telemedicine interventions as interventions that are rendered over spatial distances via information and communication technologies. In the articles we reviewed, delivery modes were ‘smartphone apps’, ‘emails’, ‘internet websites’, ‘telephone’ (alone, with an app, with an online-community, with emails or other messengers), and ‘videoconference’. The most frequent delivery mode was ‘websites’, followed by any interventions using telephone and ‘apps’. The ‘website’ and ‘telephone’ interventions were predominantly successful, whereas the results of the ‘app’ interventions were inconsistent. The ‘websites’ showed significant improvements for depression more often than no effects. Mixed results were found for postnatal depression and stress. On the other hand, anxiety was twice as likely to have no effects as significant improvements. Since both ‘websites’ and ‘apps’ are modes that operate online in a digital environment and with the use of apps being widespread and frequent nowadays, it was somewhat surprising that the ‘apps’ showed more mixed results. The use of ‘apps’ only showed a significant improvement once each for depression and postnatal depression, and otherwise showed no significant results and even a significant deterioration in one case. However, the number of studies with ‘apps’ was relatively small with *n* = 7. Apps are taking on an increasingly important role in everyday life and are available at almost every moment. They also offer a good opportunity for data collection in ecologically valid real-world environments through human behaviour sampling methods such as ecological momentary assessments [80–82]. Hence, it is important to further investigate what the necessary conditions and circumstances are so that the use of apps leads to successful results. The success of ‘telephone’ interventions can probably be attributed to the fact that ‘telemedicine’ interventions with personal contacts are commonly more successful than interventions without personal contacts [83, 84]. This is, however, not fully in line with our findings.

#### Personal contacts

The majority of interventions with personal contacts was successful (55%) yet an even higher 80% of the interventions without personal contacts were also successful. A possible explanation for this could be the degree of severity of the disease and the resulting complexity of treatment, which Zhao found to be significantly different for depressive symptoms based on the severity of PPD [85]. It is conceivable that the severity and complex issues usually require more intensive treatment and e.g. more face-to-face time or at least blended care. Blended care is a relatively recent concept that combines regular face-to-face treatments, such as therapist-led CBT sessions with telemedicine modules, such as iCBT, in order to take advantage of the benefits of both approaches while mitigating their disadvantages [86]. The PubMed database lists several articles on this subject, yet only since 2016. None of the articles in this sample used the term “blended care”. Therefore, it was not possible to consider “blended care” in this review.

The type of contact interaction was divided into ‘bilateral contacts’ (between study participants and study staff/peers), ‘forum’ (which allowed an exchange between different parties in a common forum) and a mix of both. Most ‘bilateral contacts’ were successful (60%). Similarly, the exchange in ‘forums’ seems to be a very promising approach and should be considered in upcoming studies.

### Most common mental health-related outcomes

Some mental health-related outcomes were more common than others. The most common were depression, postnatal depression, anxiety and stress, and they were therefore selected for further examination. No reliable statement could be made for the mental health-related outcomes found less frequently within this review due to the low number of studies and the differences in study quality. For depression and postnatal depression, the majority of interventions (57%) showed a significant improvement for the intervention groups. Several interventions found no significant differences between groups (40%). Just one article in the review found a significant worsening for postnatal depression (3%). For anxiety there were almost twice as many studies that found no effect than studies that found significant improvements for the intervention groups. Regarding stress, the number of articles that found a significant effect for the intervention groups is almost as high as those that found no differences between the groups. Whether anxiety was considered as a primary or secondary outcome in the studies may make a difference in the results. To further illustrate the above findings, we will discuss the findings for all four mental health-related outcomes with regard to the characteristics of the interventions and RCTs.

#### Depression

For depression, the most common intervention contents were ‘iCBT’, ‘education lessons’, ‘mindfulness’ and ‘peer support’. The intervention ‘iCBT’ predominately resulted in significant improvements, whereas ‘education lessons’, ‘mindfulness’ and ‘peer support’ showed mixed results. However, a smaller number of articles mentioned the three latter intervention contents than was the case for ‘iCBT’. The intervention types ‘self-help tool’ and ‘support’ were most common for depression. When a ‘self-help tool’ was used, there were significant improvements equally as often as no effects. Any supportive interventions (from professional staff as well as peers) showed significant improvements more often than no effects. The most common intervention target for depression was ‘symptom reduction’. There were twice as many studies in which ‘symptom reduction’ was successful than those where it was unsuccessful. In terms of delivery mode, ‘websites’ were deemed to be successful in 64% of studies, ‘telephone’ in 67% and ‘telephone mixed with emails or other messengers’ had a success rate of 100%. However, it should be noted that the latter two delivery modes combined were only *n* = 3 in total. The majority of interventions were successful when they included personal contact. All in all, interventions addressing depression were successful when they used ‘iCBT’, were ‘supportive’, targeted ‘symptom reduction’ and included personal contacts. This was consistent with other reviews that investigated web-based iCBT interventions targeting maternal mood outcomes in pregnant women and mothers in postpartum stage [26, 27, 31]. The results must be interpreted with some caution, keeping in mind that the resulting classification from the risk of bias assessment indicated that 27% of the articles either gave cause for "some concerns" or had a "high" risk of bias. Further studies are required to establish when a given approach for symptom reduction is successful and when it is not.

#### Postnatal depression

The intervention contents ‘iCBT’ and ‘education lessons’ showed mixed results. Significant improvements were successfully achieved with ‘peer support’, but the number of studies was small (*n* = 2). Regarding ‘self-help tools’ for postnatal depression more articles reported significant improvements. Studies containing ‘professional and/or peer support’ produced mixed results. ‘Treatment’ more often resulted in no effects than in significant improvements, but the number of studies was small (*n* = 2). Unlike with depression, ‘symptom reduction’ interventions had mixed results for postnatal depression. Two-thirds of the articles reported successful interventions for ‘preventing stress’. Hence, in postnatal depression, the ‘prevention approach’ and ‘support’ through peers was found to be promising. To improve self-help tool strategies, further investigation should determine in which cases a ‘self-help tool’ is successful. Appropriate delivery modes should be further investigated. A narrow majority of interventions using ‘websites’ achieved significant improvements. In the case of ‘apps’ and ‘telephone’, only a minority did so. For postnatal depression, no significant effects or significant improvements were observed, whereby an equal proportion of the studies included personal contacts. Furthermore, one article with personal contacts even observed a significant deterioration. On the other hand, ‘telephone’ mixed with ‘app’ or with ‘emails or other messengers’ were successful delivery modes, but the number of studies was very small with *n* = 2 studies in total. Again, the results must be interpreted with caution. The risk of bias assessment showed that for 43% articles there were either some concerns or there was a high risk.

#### Anxiety

Peer support was also a successful intervention for anxiety; however, the number of studies was small (*n* = 2). The intervention ‘iCBT’ showed mixed results. This finding is consistent with the findings of the systematic review by Ashford et al. [29] and the systematic review and meta-analysis by Loughnan et al. [27], whereas the meta-analytic review by Lau et al. [31] supported the efficacy of therapist-supported iCBT for improving anxiety with a small effect size (d = 0.36). Interventions with ‘education lessons’ and ‘mindfulness’ were less successful; improvements were reported in only one of five studies (education lessons) or one of three (mindfulness). ‘Self-help tools’ predominately achieved no significant effects for anxiety. ‘Support’, on the other hand, showed significantly improved effects. There were more studies targeting ‘symptom reduction’ for anxiety which had no effect than studies with significant effects, which is in line with Loughnan et al. [27]. With regard to anxiety, an approach with the goal of ‘preventing stress’ seems promising and should be investigated further. The approaches for ‘managing/coping’ and ‘to strengthen mental health’ both showed no effects, but the number of studies (*n* = 5) is also small. In terms of the delivery mode, only ‘websites’, ‘telephone’ and ‘telephone mixed with app or with emails or other messengers’ were able to achieve any significant improvements. For ‘websites’, this was only a third of the studies. All delivery modes using a ‘telephone’ were successful, but the number of studies was small with just *n* = 3. For anxiety, personal contacts did not lead to more significant improvements being reported than no effects. This seems to contradict the previous statement about the success of ‘telephone’ interventions. However, interventions addressing anxiety disorders rarely included the delivery mode ‘telephone’, and instead mostly employed ‘websites’. However, when there was a ‘telephone’ included, significant improvements were also found for anxiety. The risk of bias assessment revealed that a clear majority (87.5%) of the articles on anxiety were ranked as "low" risk.

Anxiety seems to be more difficult to approach through telemedicine. To evaluate this in more detail, we differentiated between anxiety as a primary or secondary outcome. From nine studies in total that mentioned anxiety as (one of) their primary outcome(s), just two (22%) reported a significant improvement for the intervention group, both used ‘websites’ as their intervention. Of these two, one administered ‘iCBT’ and the other employed a ‘mindfulness’ intervention; one was namely designed as a ‘self-help tool’ whereas the other one was ‘support’. Both targeted ‘symptom reduction’. In total 15 studies explored anxiety as a secondary outcome, of which six (40%) showed a significant improvement for the intervention group. Of those six, three studies used ‘websites’ and three used different communication channels, but all three included ‘telephone’. Three studies administered ‘iCBT’, one used ‘education lessons’ and two provided ‘peer support’. Four were designed as ‘self-help tools’, two as ‘support’. Three of the studies targeted ‘preventing stress/mental health impairment’, one study target was ‘not in the field of mental health’ and two targeted ‘symptom reduction’.

The findings were too heterogeneous to be able to discuss the requirements for a successful intervention. Nonetheless, tailoring telemedicine interventions specifically for anxiety is important due to the high prevalence of this particular health issue during pregnancy and the small impact reported so far [27]. Hence, further research is vital. Nevertheless, direct bilateral contact between participants and professionals seems to be important for the success of telemedicine interventions for people with anxiety disorders.

#### Stress

In terms of interventions targeting stress, ‘iCBT’ was successful (significant improvement), whereas ‘education lessons’ showed no effects more often than significantly improved effects. ‘Self-help tools’ had no effects more often than resulting in significant improvements. The number of other types of intervention was too small to enable a reliable interpretation, with only one of each (‘support’, ‘monitoring’ and ‘treatment’). For stress, the most investigated intervention target was ‘symptom reduction’. As with the other mental health-related outcomes, the results for ‘symptom reduction’ were heterogeneous. More research is also needed here. For interventions targeting stress, ‘personal contacts’ did not lead to more instances of significant improvements being reported than those with no effects. There were two delivery modes which produced some significant improvements. ‘Websites’ were 50% successful (*n* = 4). ‘Telephone’ / online community was successful, but only reported once. In conclusion, ‘iCBT’ is a successful telemedicine intervention to combat stress. This is in line with Lau et al. [31] who found a large effect (d = 0.84) on improving stress symptoms. An 80% majority of the articles were ranked as “low” risk in the risk of bias assessment.

The results showed that there is no successful concept which can be employed for all mental health problems and disorders in the target group of pregnant women and new mothers. This is not even the case when differentiating according to specific disorders, as the example of anxiety has shown. Lau et al. [31] came to a similar conclusion in their work and stated “A one-size-fits-all approach is unlikely to succeed considering the complexities and idiosyncrasies of specific health conditions”. Considering the rise of the concept of personalized medicine, further research could also take a patient-specific approach into account. Future telemedicine interventions could be designed in such a way that the intervention components and contents are explicitly adaptable to the needs of the patient (patient-individual).

### Feasibility, acceptance and implementation barriers

Feasibility, acceptance and implementation barriers were not reported in every article of this sample. Of those articles that reported feasibility and/or acceptance, the positive assessments of feasibility and acceptance clearly outweighed the negative ones.

However, a reliable conclusion cannot be drawn with this small number of statements. Nevertheless, the reported difficulties may arise in implementing other telemedicine interventions and should be considered and addressed when planning further such interventions.

### Strengths and limitations

The inclusion criteria for this review were deliberately broad in order to reflect the range of telemedicine interventions for the targeted patient groups. Hence, the variance of mental health-related outcomes, intervention types and contents was equally broad. This was a strength and a limitation at the same time. It was a strength because it reflected the broad range of telemedicine interventions in this field. This allowed for a comprehensive examination of the possibilities these kinds of interventions might have in the field of mental health problems and the targeted patient group. It was a limitation at the same time because there was considerable variation between the studies. As a consequence, the differences between the articles were large and the sample sizes for each detailed intervention commonality were small.

A strength of this review was that it was carried out according to the PRISMA guidelines [32] and using the open access online tool CADIMA [33]. CADIMA supports the whole process of conducting a systematic review. The literature search results from the databases were uploaded in CADIMA. Hence, the procedures of screening and article selection were performed systematically. The screening for the inclusion criteria was independently conducted by two reviewers (AB and US) with CADIMA. Both researchers performed several rounds of consistency training which resulted in a ‘good’ consistency (Kappa value 0.7). Another strength was that due to the large number of RCTs found, it was possible to limit the studies included to RCTs. RCTs have the greatest strength of evidence.

To critically appraise the validity of the included articles risk of bias assessments [34] were performed with the Cochrane risk-of-bias tool for randomized trials (RoB 2) according to the PRISMA guidelines [32]. A table with the risk of bias judgements is provided in the supplementary file S5. As a limitation, it must be pointed out that the risk-of-bias tool was not ideally suited to telemedicine interventions. Blinding is not usually possible with these interventions. Losses to follow up are also often higher in patients with mental impairments [27]. In addition, these are often RCTs with a pragmatic approach. In contrast to RCTs with a clinical approach, the conditions are less strict and closer to everyday care. The risk of bias can quickly increase due to the particular context of these studies (telemedicine intervention and mental health). Therefore, we have adapted the risk of bias assessment tool for our purposes and skipped the domain ‘risk of bias due to deviations from the intended interventions (effect of assignment to intervention)’. The overall risk of bias was adjudged to be ‘low’ 24 times (55%), five times there were ‘some concerns’ (11%), and the risk of bias was assessed to be ‘ high’ 14 times (32%), while there was also one article that could not be assessed due to lack of information (see supplementary file S5). The articles for which the assessment indicated ‘some concerns’ or a ‘high’ risk were mostly judged to be at risk of bias because of domain three ‘missing outcome data’ due to increased or high losses to follow up. It should be borne in mind that this is a common problem among these studies and that it does not necessarily allow conclusions to be drawn about the quality of the study.

The heterogeneity of interventions (of their delivery mode, their targets, their contents and approaches) and the lack of consistency in the addressed mental health-related outcomes (meaning not to being limited to, for example, depression), narrowed the statistical comparability, but revealed structural differences. Generalisability was also limited because most studies included only women but three studies included parental couples.

## Conclusions

The results of this review showed that telemedicine interventions are generally suitable for addressing the mental health of pregnant women and new mothers. Just one article reported a significant worsening when compared to the control group. All other articles showed either significant improvements for the telemedicine interventions or there was no significant difference between the groups. This means that telemedicine interventions are usually not an inferior option and do no harm. They are suitable for use when face-to-face care is not available, e. g. when there are no scheduling capacities, when mobility is restricted or when a pandemic occurs.

Answering the question as to which of the characteristics are successful for which mental health-related outcome is more complex. Among the four mental health-related outcomes that were most frequently found, interventions addressing depression and postpartum depression were more likely to be successful. For stress and anxiety, the results were not as consistent. For these four mental health-related outcomes, the successful intervention contents were ‘iCBT’ for depression and stress, and ‘peer support’ for postnatal depression and anxiety. ‘Support’, both professionally and through peers, seems to be promising. It seemed that anxiety was difficult to approach through telemedicine. Future studies on telemedicine interventions for anxiety should therefore focus on support. Aside from the ‘modern’ way of delivering interventions through websites, the good old telephone was successful for interventions targeting anxiety. Although the number of studies was small, the use of the telephone seems to be a suitable delivery mode for interventions related to anxiety. Preventive approaches appear to be promising avenues to pursue for postnatal depression and anxiety and should be investigated further to produce more reliable results. For the less frequently occurring mental health-related outcomes within this review, like post-traumatic stress disorder, wellbeing, or emotion regulation/psychological flexibility, the majority of studies reported significant improvements. However, because the number of studies examining those particular outcomes was very small (ranging between 1 – 2) no reliable statement can be made. However, it would be misleading to conclude that telemedicine interventions would only be worthwhile for depression and postnatal depression.

There is no one-size-fits-all approach to all mental health-related outcomes. To gain reliable evidence on telemedicine interventions in the field of mental health for pregnant women and new mothers, there is a need for more research that explores the different conditions, contents, modalities, targets, approaches, influences of confounders like symptom severity and co-morbidities for different mental health disorders. In doing this research, one of the challenges will be how to achieve sufficient sample sizes despite the difficulties in the recruitment and adherence behaviour among patients with mental health problems.

## Supplementary Information

**Additional file 1**. Telemedical interventions for mentally stressed, pregnant women and young mothers: protocol for a systematic literature review.

**Additional file 2:** **Supplementary file S2**. Search terms Pubmed. **Table 2**. Search term Cochrane. **Table 3**. Search term Isi web of science. **Table 4**. Search term PsychInfo.

**Additional file 3:** **Supplementary file S3.** N = 44 reports (solely articles) included in this review.

**Additional file 4: Supplementary file S4. **Mental health-related outcome findings and operationalized by measuring instruments

**Additional file 5:** **Supplementary file S5.** Risk of bias judgement.

## Data Availability

The datasets used and/or analysed during the current study are available from the corresponding author on reasonable request.

## Abbreviations

AB: Angelika Beyer (author)
ACT: Acceptance and Commitment Therapy
CADIMA: Central Access Database for Impact Assessment
iBA: Internet-based Behavioural Activation
iCBT: Internet-delivered Cognitive Behavioural Therapy
iCMT: Internet-delivered Compassionate Mind Training
MeSH: Medical Subject Headings
PICO: Population Intervention Comparator Outcome
PRISMA: Preferred Reporting Items for Systematic and Meta-Analyses
PROSPERO: International Prospective Register of Systematic Reviews
RCT: Randomized Controlled Trial
RoB: Risk of Bias
TAU: Treatment As Usual
US: Ulrike Stentzel (author)
