# Supplementary material for: Mental health-related telemedicine interventions for pregnant women and new mothers: a systematic literature review
Source: BMC Psychiatry. 2023 Apr 28;23:292. doi: 10.1186/s12888-023-04790-0 (PMC10148488; doi:10.1186/s12888-023-04790-0)
Supplement: Supplementary file 2 — Additional file 2: Supplementary file S2. Search terms Pubmed. Table 2. Search term Cochrane. Table 3. Search term Isi web of science. Table 4. Search term PsychInfo. [file 12888_2023_4790_MOESM2_ESM.docx]

Supplementary file S2: Search terms Pubmed

| **ID** | **Date** | **Search Strategy** | **Limiters/Filters** | **# of Results** |
| --- | --- | --- | --- | --- |
| Pubmed # A | 16.11.2020 | Search: **(((((((((((((((((telemedicine[Title/Abstract]) OR "tele psychiatry" [Title/Abstract]) OR web [Title/Abstract]) OR apps[Title/Abstract]) OR applications [Title/Abstract]) OR tablet[Title/Abstract]) OR smartphone[Title/Abstract]) OR "text messages"[Title/Abstract]) OR "phone call"[Title/Abstract]) OR mhealth[Title/Abstract]) OR eHealth[Title/Abstract]) OR telepsychiatry[Title/Abstract]) OR telehealth[Title/Abstract]) OR "Mobile Health"[Title/Abstract]) OR "tele care"[Title/Abstract]) OR telemedical[Title/Abstract]) OR internet[Title/Abstract]) AND (((((((((((((("mentally stressed"[Title/Abstract]) OR "mental health"[Title/Abstract]) OR "mental disorder"[Title/Abstract]) OR "mental disease"[Title/Abstract]) OR "mentally ill"[Title/Abstract]) OR psychological[Title/Abstract]) OR "psychological impairment"[Title/Abstract]) OR "psychologically affected"[Title/Abstract]) OR "psychologically stressed"[Title/Abstract]) OR "psychological disorder"[Title/Abstract]) OR "psychological distress"[Title/Abstract]) OR "psychiatric disorder"[Title/Abstract]) OR "psychiatric disease"[Title/Abstract]) OR depression[Title/Abstract]) AND ((((((((((pregnancy[Title/Abstract]) OR pregnant[Title/Abstract]) OR prenatal[Title/Abstract]) OR prepartal[Title/Abstract]) OR antenatal[Title/Abstract]) OR prepartum[Title/Abstract]) OR peripartum[Title/Abstract]) OR gestation[Title/Abstract]) OR gravidic[Title/Abstract]) OR childbearing[Title/Abstract])** Filters: **from 2007/1/1 - 3000/12/12** | *Filters applied: From 2007/1/1 to 3000/12/12* | 490 |
| Pubmed # B | 16.11.2020 | Search: **(((((((((((((((((telemedicine[Title/Abstract]) OR "tele psychiatry" [Title/Abstract]) OR web [Title/Abstract]) OR apps[Title/Abstract]) OR applications[Title/Abstract]) OR tablet[Title/Abstract]) OR smartphone[Title/Abstract]) OR "text messages"[Title/Abstract]) OR "phone call"[Title/Abstract]) OR mhealth[Title/Abstract]) OR eHealth[Title/Abstract]) OR telepsychiatry[Title/Abstract]) OR telehealth[Title/Abstract]) OR "Mobile Health"[Title/Abstract]) OR "tele care"[Title/Abstract]) OR telemedical[Title/Abstract]) OR internet[Title/Abstract]) AND (((((((((((((("mentally stressed"[Title/Abstract]) OR "mental health"[Title/Abstract]) OR "mental disorder"[Title/Abstract]) OR "mental disease"[Title/Abstract]) OR "mentally ill"[Title/Abstract]) OR psychological[Title/Abstract]) OR "psychological impairment"[Title/Abstract]) OR "psychologically affected"[Title/Abstract]) OR "psychologically stressed"[Title/Abstract]) OR "psychological disorder"[Title/Abstract]) OR "psychological distress"[Title/Abstract]) OR "psychiatric disorder"[Title/Abstract]) OR "psychiatric disease"[Title/Abstract]) OR depression[Title/Abstract]) AND (((((((((("young mothers"[Title/Abstract]) OR "new mums"[Title/Abstract]) OR "new moms"[Title/Abstract]) OR "young mums"[Title/Abstract]) OR "young moms"[Title/Abstract]) OR peripartum[Title/Abstract]) OR postnatal[Title/Abstract]) OR postpartal[Title/Abstract]) OR postpartum[Title/Abstract]) OR Puerperium[Title/Abstract])** Filters: **from 2007/1/1 - 3000/12/12** | *Filters applied: From 2007/1/1 to 3000/12/12* | 343 |

Table 2: Search term Cochrane

| **ID** | **#** | **Date** | **Search Strategy** | **Limiters/Filters** | **details** | **# of Results** |
| --- | --- | --- | --- | --- | --- | --- |
| Cochrane # A (Trials) | | 16.11.2020 | 593 Trials matching "#1 - ("telemedicine" OR "tele psychiatry" OR "telepsychiatry" OR "tele-psychiatry" OR "web" OR "apps" OR "application" OR "tablet" OR "smartphone" OR "text messages" OR "phone call" OR "mhealth" OR "eHealth" OR "telehealth" OR "Mobile Health" OR "tele care" OR "telemedical" OR "internet"):ti,ab,kw AND ("mentally stressed" OR "mental health" OR "mental disorder" OR "mental disease" OR "mentally ill" OR "psychological" OR "psychological impairment" OR "psychologically affected" OR "psychologically stressed" OR "psychological disorder" OR "psychological distress" OR "psychiatric disorder" OR "psychiatric disease" OR "depression"):ti,ab,kw AND ("pregnancy" OR "pregnant" OR "prenatal" OR "prepartal" OR "antenatal" OR "prepartum" OR "peripartum" OR "gestation" OR "gravidic" OR "childbearing"):ti,ab,kw" with Cochrane Library publication date from Jan 2007 to present | with Cochrane Library publication date from Jan 2007 to present | Trials | 593 |
| Cochrane # B (Trials) | | 16.11.2020 | 284 Trials matching "#2 - ("telemedicine" OR "tele psychiatry" OR "web " OR "application" OR "apps" OR "tablet" OR "smartphone" OR "text messages" OR "phone call" OR "mhealth" OR "eHealth" OR "telepsychiatry" OR "telehealth" OR "Mobile Health" OR "tele care" OR "telemedical" OR "internet"):ti,ab,kw AND ("mentally stressed" OR "mental health" OR "mental disorder" OR "mental disease" OR "mentally ill" OR "psychological" OR "psychological impairment" OR "psychologically affected" OR "psychologically stressed" OR "psychological disorder" OR "psychological distress" OR "psychiatric disorder" OR "psychiatric disease" OR "depression"):ti,ab,kw AND ("young mothers" OR "new mums" OR "new moms" OR "young mums" OR "young moms" OR "peripartum" OR "postnatal" OR "postpartal" OR "postpartum" OR "Puerperium"):ti,ab,kw" with Cochrane Library publication date from Jan 2007 to present | with Cochrane Library publication date from Jan 2007 to present | Trials | 284 |

Table 3: Search term Isi web of science

| **ID Endnote** | **#** | **Date** | **Search Strategy** | **Limiters/Filters** | **# of Results** |
| --- | --- | --- | --- | --- | --- |
| ISI Web of Science # A | #1 | 16.11.2020 | (TS=(telemedicine OR tele psychiatriy OR web OR app OR applications OR tablet OR smartphone OR text messages OR phone call OR mhealth OR eHealth OR telepsychiatry OR telehealth OR Mobile Health OR tele care OR telemedical OR internet) )  AND  LANGUAGE:  (English) AND DOCUMENT  TYPES:  (Article)  Indexes=SCI-EXPANDED, SSCI, A&HCI, CPCI-S, CPCI-SSH, BKCI-S, BKCI-SSH, ESCI, CCR-EXPANDED, IC Timespan=2007-2020 |  | 2.229.162 |
|  | #2 | 16.11.2020 | (TS=(mentally stressed OR mental health OR mental disorder OR mental disease OR mentally ill OR psychological OR psychological impairment OR psychologically affected OR psychologically stressed OR psychological disorder OR psychological distress OR psychiatric disorder OR psychiatric disease OR depression) )  AND  LANGUAGE:  (English) AND DOCUMENT  TYPES:  (Article)  Indexes=SCI-EXPANDED, SSCI, A&HCI, CPCI-S, CPCI-SSH, BKCI-S, BKCI-SSH, ESCI, CCR-EXPANDED, IC Timespan=2007-2020 |  | 556.295 |
|  | #3 | 16.11.2020 | (TS=(pregnancy OR pregnant OR prenatal OR prepartal OR antenatal OR prepartum OR peripartum OR gestation OR gravidic OR childbearing) )  AND  LANGUAGE:  (English) AND DOCUMENT  TYPES:  (Article)  Indexes=SCI-EXPANDED, SSCI, A&HCI, CPCI-S, CPCI-SSH, BKCI-S, BKCI-SSH, ESCI, CCR-EXPANDED, IC Timespan=2007-2020 |  | 266.298 |
|  | #4 | 16.11.2020 | #3 AND #2 AND #1  Indexes=SCI-EXPANDED, SSCI, A&HCI, CPCI-S, CPCI-SSH, BKCI-S, BKCI-SSH, ESCI, CCR-EXPANDED, IC Timespan=2007-2020 |  | 686 |
|  |  |  |  |  |  |
|  |  |  |  |  |  |
|  |  |  |  |  |  |
| ISI Web of Science # B | #1 | 16.11.2020 | (TS=(telemedicine OR tele psychiatriy OR web OR app OR applications OR tablet OR smartphone OR text messages OR phone call OR mhealth OR eHealth OR telepsychiatry OR telehealth OR Mobile Health OR tele care OR telemedical OR internet) )  AND  LANGUAGE:  (English) AND DOCUMENT  TYPES:  (Article)  Indexes=SCI-EXPANDED, SSCI, A&HCI, CPCI-S, CPCI-SSH, BKCI-S, BKCI-SSH, ESCI, CCR-EXPANDED, IC Timespan=2007-2020 |  | 2.229.162 |
|  | #2 | 16.11.2020 | (TS=(mentally stressed OR mental health OR mental disorder OR mental disease OR mentally ill OR psychological OR psychological impairment OR psychologically affected OR psychologically stressed OR psychological disorder OR psychological distress OR psychiatric disorder OR psychiatric disease OR depression) )  AND  LANGUAGE:  (English) AND DOCUMENT  TYPES:  (Article)  Indexes=SCI-EXPANDED, SSCI, A&HCI, CPCI-S, CPCI-SSH, BKCI-S, BKCI-SSH, ESCI, CCR-EXPANDED, IC Timespan=2007-2020 |  | 556.295 |
|  | #5 | 16.11.2020 | (TS=(young mothers OR new mums OR new moms OR young mums OR young moms OR peripartum OR postnatal OR postpartal OR postpartum OR Puerperium) )  AND  LANGUAGE:  (English) AND DOCUMENT  TYPES:  (Article)  Indexes=SCI-EXPANDED, SSCI, A&HCI, CPCI-S, CPCI-SSH, BKCI-S, BKCI-SSH, ESCI, CCR-EXPANDED, IC Timespan=2007-2020 | Language: English document types: Articles Timespan: 2007 - 2019 | 100.297 |
|  | #6 | 16.11.2020 | #5 AND #2 AND #1  Indexes=SCI-EXPANDED, SSCI, A&HCI, CPCI-S, CPCI-SSH, BKCI-S, BKCI-SSH, ESCI, CCR-EXPANDED, IC Timespan=2007-2020 |  | 617 |

Table 4_ Search term PsychInfo

| **ID** | **Date** | **Search Strategy** | **Limiters/Filters** | **# of Results** |
| --- | --- | --- | --- | --- |
| Psycinfo #A | 16.11.2020 | ( "telemedicine" OR "tele psychiatry" OR "telepsychiatry" OR "tele-psychiatry" OR "web" OR "apps" OR "application" OR "tablet" OR "smartphone" OR "text messages" OR "phone call" OR "mhealth" OR "eHealth" OR "telehealth" OR "Mobile Health" OR "tele care" OR "telemedical" OR "internet" ) AND ( "mentally stressed" OR "mental health" OR "mental disorder" OR "mental disease" OR "mentally ill" OR "psychological" OR "psychological impairment" OR "psychologically affected" OR "psychologically stressed" OR "psychological disorder" OR "psychological distress" OR "psychiatric disorder" OR "psychiatric disease" OR "depression" ) AND ( "pregnancy" OR "pregnant" OR "prenatal" OR "prepartal" OR "antenatal" OR "prepartum" OR "peripartum" OR "gestation" OR "gravidic" OR "childbearing" ) | 2007 - 2020 | 666 |
| Psycinfo #B | 16.11.2020 | ( "telemedicine" OR "tele psychiatry" OR "telepsychiatry" OR "tele-psychiatry" OR "web" OR "apps" OR "application" OR "tablet" OR "smartphone" OR "text messages" OR "phone call" OR "mhealth" OR "eHealth" OR "telehealth" OR "Mobile Health" OR "tele care" OR "telemedical" OR "internet" ) AND ( "mentally stressed" OR "mental health" OR "mental disorder" OR "mental disease" OR "mentally ill" OR "psychological" OR "psychological impairment" OR "psychologically affected" OR "psychologically stressed" OR "psychological disorder" OR "psychological distress" OR "psychiatric disorder" OR "psychiatric disease" OR "depression" ) AND ("young mothers" OR "new mums" OR "new moms" OR "young mums" OR "young moms" OR "peripartum" OR "postnatal" OR "postpartal" OR "postpartum" OR "Puerperium") | 2007 - 2020 | 357 |
