# Supplementary material for: Mental health-related telemedicine interventions for pregnant women and new mothers: a systematic literature review
Source: BMC Psychiatry. 2023 Apr 28;23:292. doi: 10.1186/s12888-023-04790-0 (PMC10148488; doi:10.1186/s12888-023-04790-0)
Supplement: Supplementary file 3 — Additional file 3: Supplementary file S3. N = 44 reports (solely articles) included in this review. [file 12888_2023_4790_MOESM3_ESM.docx]

Supplementary file S3: N = 44 reports (solely articles) included in this review

| **Study-ID** | **Author** | **Publication year** | **Title** | **Reference** |
| --- | --- | --- | --- | --- |
| #01 | Azza H. Ahmed, Ali M. Roumani, Kinga Szucs, Lingsong Zhang, Demetra King, | 2016 | The effect of interactive Web-based monitoring on breastfeeding exclusivity, intensity, and duration in healthy term infants after hospital discharge | [42] |
| #02 | Abby D. Altazan, Leanne M. Redman, Jeffrey H. Burton, Robbie A. Beyl, Loren E. Cain, Elizabeth F. Sutton and Corby K. Martin | 2019 | Mood and quality of life changes in pregnancy and postpartum and the effect of a behavioral intervention targeting excess gestational weight gain in women with overweight and obesity: a parallel-arm randomized controlled pilot trial | [35] |
| #03 | Alinne Z. Barrera, Robert E. Wickham, and Ricardo F. Muñoz | 2015 | Online prevention of postpartum depression for Spanish- and English-speaking pregnant women: A pilot randomized controlled trial | [43] |
| #04 | Kelly A. Bennion, Deborah Tate, Karen Muñoz-Christian, and Suzanne Phelan | 2020 | Impact of an Internet-Based Lifestyle Intervention on Behavioral and Psychosocial Factors During Postpartum Weight Loss | [44] |
| #05 | Yvonne S. Butler Tobah, Annie LeBlanc, Megan E. Branda, Jonathan W. Inselman, BS, Megan A. Morris, Jennifer L. Ridgeway, Dawn M. Finnie, Regan Theiler, Vanessa E. Torbenson, Ellen M. Brodrick, Marnie Meylor de Mooij, Bobbie Gostout, Abimbola Famuyide | 2019 | Randomized comparison of a reduced-visit prenatal care model enhanced with remote monitoring | [45] |
| #06 | Claudia Carissoli, Daniela Villani, Deborah Gasparri and Giuseppe Riva, | 2017 | Enhancing psychological wellbeing of women approaching the childbirth: a controlled study with a mobile application | [56] |
| #07 | Ko Ling Chan, Wing Cheong Leung, Agnes Tiwari, Ka Lun Or, Patrick Ip | 2019 | Using Smartphone-Based Psychoeducation to Reduce Postnatal Depression Among First-Time Mothers: Randomized Controlled Trial | [36] |
| #08 | C.-L. Dennis, E. Hodnett, Heather M. Reisman, L. Kenton, J. Weston, J. Zupancic, D. E. Stewart, Lillian Love, A. Kiss | 2009 | Effect of peer support on prevention of postnatal depression among high risk women: multisite randomised controlled trial | [46] |
| #09 | Tracy A. Dennis-Tiwary, Samantha Denefrio, Shari Gelber | 2017 | Salutary Effects of an Attention Bias Modification Mobile Application on Biobehavioral Measures of Stress and Anxiety during Pregnancy | [47] |
| #10 | Jennifer Duffecy, Rebecca Grekin, Hannah Hinkel, Nicholas Gallivan, Graham Nelson, MA, Michael W O'Hara | 2019 | A Group-Based Online Intervention to Prevent Postpartum Depression (Sunnyside): Feasibility Randomized Controlled Trial | [48] |
| #11 | Ana Fonseca, Fabiana Monteiro, Stephanie Alves, Ricardo Gorayeb, Maria Cristina Canavarro | 2019 | Be a Mom, a Web-Based Intervention to Prevent Postpartum Depression: The Enhancement of Self-Regulatory Skills and Its Association With Postpartum Depressive Symptoms | [58] |
| #12 | Ana Fonseca, Stephanie Alves, Fabiana Monteiro, Ricardo Gorayeb, Maria Cristina Canavarro | 2020 | Be a Mom, a Web-Based Intervention to Prevent Postpartum Depression: Results From a Pilot Randomized Controlled Trial | [57] |
| #13 | Erik Forsell, Marie Bendix, Fredrik Holländare, Barbara Szymanska von Schultz, Josefine Nasiell, Margareta Blomdahl-Wetterholm, Caroline Eriksson, Sara Kvarned, Johanna Lindau van der Linden, Elin Söderberg, Jussi Jokinen, Katarina Wide, Viktor Kaldo | 2017 | Internet delivered cognitive behavior therapy for antenatal depression: A randomised controlled trial | [20] |
| #14 | Isobel Gammer, Charlotte Hartley-Jones, Fergal W. Jones | 2020 | A Randomized Controlled Trial of an Online, Compassion-Based Intervention for Maternal Psychological Well-Being in the First Year Postpartum | [59] |
| #15 | Dwenda Kay Gjerdingen, Patricia McGovern, Rebekah Pratt, Linda Johnson, and Scott Crow | 2013 | Postpartum Doula and Peer Telephone Support for Postpartum Depression: A Pilot Randomized Controlled Trial | [49] |
| #16 | Lu Guo, Jing Zhang, Liping Mu, Zhao Ye | 2020 | Preventing Postpartum Depression With Mindful Self-Compassion Intervention - A Randomized Control Study | [65] |
| #17 | Silje Marie Haga, Filip Drozd, Carina Lisøy, Tore Wentzel-Larsen, Kari Slinning | 2018 | Mamma Mia – A randomized controlled trial of an internet-based intervention for perinatal depression | [37] |
| #18 | Silje Marie Haga, Patricia Kinser, Tore Wentzel-Larsen, Carina Lisøy, Susan Garthus-Niegel, Kari Slinning & Filip Drozd | 2020 | Mamma Mia – A randomized controlled trial of an internet intervention to enhance subjective well-being in perinatal women | [74] |
| #19 | Hanna M Heller, Adriaan W Hoogendoorn, Adriaan Honig, Birit F. P. Broekman, Annemieke van Straten | 2020 | The Effectiveness of a Guided Internet-Based Tool for the Treatment of Depression and Anxiety in Pregnancy (MamaKits Online): Randomized Controlled Trial | [38] |
| #20 | A.G. Ishola and J. Chipps | 2015 | The use of mobile phones to deliver acceptance and commitment therapy in the prevention of mother–child HIV transmission in Nigeria | [73] |
| #21 | Nana Jiao, Lixia Zhu, Yap Seng Chong, Wai-Chi Sally Chan, Nan Luo, Wenru Wang, Rongfang Hu, Yiong Huak Chan, Hong-Gu He | 2019 | Web-based versus home-based postnatal psychoeducational interventions for first-time mothers: A randomised controlled trial | [75] |
| #22 | David A. Kalmbach, Philip Cheng, Louise M. O'Brien, Leslie M. Swanson, Roopina Sangha, Srijan Sen, Constance Guille, Andrea Cuamatzi-Castelan, Alasdair L. Henry, Thomas Roth, Christopher L. Drake | 2020 | A randomized controlled trial of digital cognitive behavioral therapy for insomnia in pregnant women | [50] |
| #23 | Alex R. Kelman, Benjamin S. Evare, Alinne Z. Barrera, Ricardo F. Muñoz, Paul Gilbert | 2017 | A proof‐of‐concept pilot randomized comparative trial of brief Internet‐based compassionate mind training and cognitivebehavioral therapy for perinatal and intending to become pregnant women | [76] |
| #24 | Adele Krusche, Maret Dymond, Susannah E. Murphy, Catherine Crane | 2018 | Mindfulness for pregnancy: A randomised controlled study of online mindfulness during pregnancy | [60] |
| #25 | Siobhan A. Loughnan, Amanda Sie, Megan J. Hobbs, Amy E. Joubert, Jessica Smith, Hila Haskelberg, Alison E.J. Mahoney, Natalie Kladnitski, Christopher J. Holt, Jeannette Milgrom, Marie-Paule Austin, Gavin Andrews, Jill M Newby | 2019 | A randomized controlled trial of ‘MUMentum Pregnancy’: Internet-delivered cognitive behavioral therapy program for antenatal anxiety and depression | [39] |
| #26 | Siobhan A. Loughnan, Christine Butler, Amanda A. Sie, Ashlee B. Grierson, Aileen Z. Chen, Megan J. Hobbs, Amy E. Joubert, Hila Haskelberg, Alison Mahoney, Christopher Holt, Alan W. Gemmill, Jeannette Milgrom, Marie-Paule Austin, Gavin Andrews, Jill M. Newby | 2019 | A randomised controlled trial of ‘MUMentum postnatal’: Internet-delivered cognitive behavioural therapy for anxiety and depression in postpartum women | [70] |
| #27 | Jeannette Milgrom, Brian G Danaher, Alan W Gemmill, Charlene Holt, Christopher J Holt, John R Seeley, Milagra S Tyler, Jessica Ross, Jennifer Ericksen | 2016 | Internet Cognitive Behavioral Therapy for Women With Postnatal Depression: A Randomized Controlled Trial of MumMoodBooster | [71] |
| #28 | Fabiana Monteiro, Marco Pereira , Maria Cristina Canavarro and Ana Fonseca | 2020 | Be a Mom’s Ecacy in Enhancing Positive Mental Health among PostpartumWomen Presenting Low Risk for Postpartum Depression: Results from a Pilot Randomized Trial | [61] |
| #30 | Katri Nieminen, Ida Berg, Katri Frankenstein, Lina Viita, Kamilla Larsson, Ulrika Persson, Loviisa Spånberger, Anna Wretman, Kristin Silfvernagel, Gerhard Andersson & Klaas Wijma | 2016 | Internet-provided cognitive behaviour therapy of posttraumatic stress symptoms following childbirth—a randomized controlled trial | [62] |
| #29 | Fei-Wan Ngai, Paul Wai-Ching Wong, Kwok-Yin Leung, Pui-Hing Chau, Ka-Fai Chung | 2015 | The Effect of Telephone-Based Cognitive-Behavioral Therapy on Postnatal Depression: A Randomized Controlled Trial | [66] |
| #31 | Heather A. O'Mahen, Joanne Woodford, Julia McGinley, Fiona C. Warren, David A Richards, Thomas R. Lynch, Rod S. Taylor | 2013 | Internet-based behavioral activation—Treatment for postnatal depression (Netmums): A randomized controlled trial | [63] |
| #32 | Bobbie Posmontier, Richard Neugebauer, Scott Stuart, Jesse Chittams,  Rita Shaughnessy | 2016 | Telephone-Administered Interpersonal Psychotherapy by Nurse-Midwives for Postpartum Depression | [51] |
| #33 | Nicole E. Pugh, Heather D. Hadjistavropoulos, Dale Dirkse | 2016 | A Randomised Controlled Trial of Therapist-Assisted, Internet-Delivered Cognitive Behavior Therapy for Women with Maternal Depression | [52] |
| #34 | Alyssa Sawyer, Amy Kaim, Huynh-Nhu Le, Denise McDonald, Murthy Mittinty, John Lynch, Michael Sawyer | 2019 | The Effectiveness of an App-Based Nurse-Moderated Program for New Mothers With Depression and Parenting Problems (eMums Plus): Pragmatic Randomized Controlled Trial | [72] |
| #35 | S. Scherer, J. Alder, J. Gaab, T. Berger, K. Ihde, C. Urech | 2016 | Patient satisfaction and psychological well-being after internet-based cognitive behavioral stress management (IB-CBSM) for women with preterm labor: A randomized controlled trial | [64] |
| #36 | Lisa B. Sheeber, John R. Seeley, Edward G. Feil, Betsy Davis, Erik Sorensen, Derek B. Kosty, Peter M. Lewinsohn | 2012 | Development and Pilot Evaluation of an Internet-Facilitated Cognitive-Behavioral Intervention for Maternal Depression | [41] |
| #37 | Shefaly Shorey , Ying/Y. Lau, Cindy-Lee Dennis, Yah Shih Chan, Wilson W.S. Tam & Yiong Huak Chan | 2017 | A randomized-controlled trial to examine the effectiveness of the ‘Home-but not Alone’ mobile-health application educational programme on parental outcomes | [68] |
| #38 | Shefaly Shorey, Cornelia Yin Ing Chee, Esperanza Debby Ng, Ying Lau, Cindy-Lee Dennis, Yiong Huak Chan | 2019 | Evaluation of a Technology-Based Peer-Support Intervention Program for Preventing Postnatal Depression (Part 1): Randomized Controlled Trial | [67] |
| #39 | Shefaly Shorey, Yvonne Peng Mei Ng, Esperanza Debby Ng, An Ling Siew, Evalotte Mörelius, Joanne Yoong, Mihir Gandhi | 2019 | Effectiveness of a Technology-Based Supportive Educational Parenting Program on Parental Outcomes (Part 1): Randomized Controlled Trial | [77] |
| #40 | Robyn Stremler, Ellen Hodnett, Laura Kenton, Kathryn Lee, Shelly Weiss, Julie Weston, Andrew Willan | 2013 | Effect of behavioural-educational intervention on sleep for primiparous women and their infants in early postpartum: multisite randomised controlled trial | [53] |
| #41 | Corinne Urech, Sandra Scherer, Martina Emmenegger, Jens Gaab, Sibil Tschudin, Irène Hoesli, Thomas Berger, Judith Alder | 2017 | Efficacy of an internet-based cognitive behavioral stress management training in women with idiopathic preterm labor: A randomized controlled intervention study | [40] |
| #42 | Lori Wozney, Janine Olthuis, Patricia Lingley-Pottie, Patrick J. McGrath, William Chaplin, Frank Elgar, Brianna Cheney, Anna Huguet, Karen Turner, Jillian Kennedy | 2017 | Strongest Families™ Managing Our Mood (MOM): a randomized controlled trial of a distance intervention for women with postpartum depression | [54] |
| #43 | Rebecca Yang, Simone N. Vigod, Jennifer M. Hensel | 2019 | Optional Web-Based Videoconferencing Added to Office-Based Care for Women Receiving Psychotherapy During the Postpartum Period: Pilot Randomized Controlled Trial | [69] |
| #44 | Mengye Yang, Ge Jia, Shiwen Sun, Cuiwei Ye, Rong Zhang, Xiaoyan Yu, | 2019 | Effects of an Online Mindfulness Intervention Focusing on Attention Monitoring and Acceptance in Pregnant Women: A Randomized Controlled Trial | [55] |
