# Supplementary material for: Mental health-related telemedicine interventions for pregnant women and new mothers: a systematic literature review
Source: BMC Psychiatry. 2023 Apr 28;23:292. doi: 10.1186/s12888-023-04790-0 (PMC10148488; doi:10.1186/s12888-023-04790-0)
Supplement: Supplementary file 4 — Additional file 4: Supplementary file S4. Mental health-related outcome findings and operationalized by measuring instruments [file 12888_2023_4790_MOESM4_ESM.docx]

Supplementary file S4: Mental health-related outcome findings and operationalized by measuring instruments

| **Mental health-related outcome** | **Measurement** |
| --- | --- |
| Postnatal Depression | Diagnostic with SCID-I |
|  | Edinburgh Postnatal Depression Scale (EPDS) |
|  | Postnatal Negative Thoughts Questionnaire (PNTQ) |
|  | Postpartum Depression Predictors Inventory-Revised (PDPI-R) |
| Anxiety | Beck Anxiety Inventory (BAI) |
|  | Depression, Anxiety and Stress Scales-Short Form (DASS) |
|  | General Anxiety Disorder 7-item scale (GAD-7) |
|  | Generalized Anxiety Disorder-2 (GAD-2) |
|  | Hamilton Anxiety Scale (HAM-A) |
|  | Hospital Anxiety and Depression Scale (HADS) |
|  | Inventory of Depression and Anxiety Symptoms (IDAS) |
|  | Pregnancy Related Anxiety Test (PRAT) |
|  | Patient Health Questionnaire-4 (PHQ-4) |
|  | State-Trait Anxiety Inventory (STAI) |
| Depression | Beck Depression Inventory II (BDI-II) |
|  | Center for Epidemiologic Studies-Depression Scale (CES-D)/ |
|  | Depression, Anxiety and Stress Scales-Short Form (DASS) |
|  | Diagnostic with SCID-I |
|  | Hamilton Depression Rating Scale (HDRS) |
|  | Hamilton Rating Scale for Depression (HAM-D) |
|  | Inventory of Depression and Anxiety Symptoms (IDAS) |
|  | Major Depressive Episode Screener (MDE-Screener) |
|  | Montgomery Åsberg Depression Rating Scale Self-report version (MADRS-S) |
|  | Patient Health Questionnaire-4 (PHQ-4) |
|  | Patient Health Questionnaire-8 (PHQ-8) |
|  | Patient Health Questionnaire-9 (PHQ-9) |
| Stress | Depression, Anxiety and Stress Scales-Short Form (DASS) |
|  | Kessler 10-item Psychological Distress scale (K-10) |
|  | Parental Stress Scale (PSS) |
|  | Parenting Stress Index (PSI) |
|  | Parenting Stress Index-Short Form (PSI-SF) |
|  | Perceived Stress Scale (PSS) |
|  | PreNatal Maternal Stress survey |
|  | Tilburg Pregnancy Distress Scale (TPDS) |
| Mindfulness AND/OR Self-Compassion | Five Facets of Mindfulness Questionnaire (FFMQ) |
|  | Mindfulness Attention Awareness Scale (MAAS) |
|  | Self-Compassion Scale (SCS) |
|  | Self-Compassion Scale Short Form (SCS-SF) |
| Wellbeing | Positive and Negative Affect Schedule (PANAS) |
|  | Psychological Wellbeing questionnaire (PWB) |
|  | Satisfaction with Life Scale (SWLS) |
|  | Warwick-Edinburgh Mental Well-being Scale (WEMWBS) |
|  | Well-Being Index World Health Organization Five (WHO-5) |
| Emotion regulation AND/OR psychological flexibility | Action and Acceptance Questionnaire (AAQ-II) |
|  | Emotion Regulation Scale [DERSSF] |
| Quality of life | EuroQol (EQ-5D) |
|  | EuroQol (EQ-5D-3L) |
|  | Quality of Life Inventory (QOLI) |
|  | Short Form Health Survey (SF-12) |
|  | World Health Organisation Quality of Life (WHOQOL-BREF) |
| Positive Mental Health | Global Assessment of Functioning (GAF) |
|  | Mental Health Continuum Short Form (MHC-SF) |
| Post-traumatic Stress disorder | Impact of Event Scale—Reversed (IES-R) |
|  | Traumatic Event Scale (TES) |
