# Supplementary material for: Mental health-related telemedicine interventions for pregnant women and new mothers: a systematic literature review
Source: BMC Psychiatry. 2023 Apr 28;23:292. doi: 10.1186/s12888-023-04790-0 (PMC10148488; doi:10.1186/s12888-023-04790-0)
Supplement: Supplementary file 5 — Additional file 5: Supplementary file S5. Risk of bias judgement. [file 12888_2023_4790_MOESM5_ESM.docx]

Supplementary file S5: Risk of bias judgement

| **Authors** | **Title** | **Reference-ID in paper** | **D1** | **D2*** | **D3** | **D4** | **D5** | **Overall** |
| --- | --- | --- | --- | --- | --- | --- | --- | --- |
| Ahmed et al. (2016) | The effect of interactive Web-based monitoring on breastfeeding exclusivity, intensity, and duration in healthy term infants after hospital discharge | [42] | Low |  | Low | Low | Low | Low |
| Altazan et al. (2019) | Mood and quality of life changes in pregnancy and postpartum and the effect of a behavioral intervention targeting excess gestational weight gain in women with overweight and obesity: a parallel-arm randomized controlled pilot trial | [35] | Low |  | Low | Low | Low | Low |
| Barrera et al. (2015) | Online prevention of postpartum depression for Spanish- and English-speaking pregnant women: A pilot randomized controlled trial | [43] | Low |  | Low | Low | Low | Low |
| Bennion et al. (2020) | Impact of an Internet-Based Lifestyle Intervention on Behavioral and Psychosocial Factors During Postpartum Weight Loss | [44] | Low |  | Low | Low | Low | Low |
| Butler Tobah et al. (2019) | Randomized comparison of a reduced-visit prenatal care model enhanced with remote monitoring | [45] | Low |  | Low | Low | Low | Low |
| Carissoli et al. (2017) | Enhancing psychological wellbeing of women approaching the childbirth: a controlled study with a mobile application | [56] | No information |  | No information | No information | No information | No information |
| Chan et al. (2019) | Using Smartphone-Based Psychoeducation to Reduce Postnatal Depression Among First-Time Mothers: Randomized Controlled Trial | [36] | Low |  | Some concerns | Low | Low | Some concerns |
| Dennis et al. (2009) | Effect of peer support on prevention of postnatal depression among high risk women: multisite randomised controlled trial | [46] | Low |  | Low | Low | Low | Low |
| Dennis-Tiwary et al. (2017) | Salutary Effects of an Attention Bias Modification Mobile Application on Biobehavioral Measures of Stress and Anxiety during Pregnancy | [47] | Low |  | Low | Low | Low | Low |
| Duffecy et al. (2019) | A Group-Based Online Intervention to Prevent Postpartum Depression (Sunnyside): Feasibility Randomized Controlled Trial | [48] | Low |  | High | Low | Low | High |
| Fonseca et al. (2019) | Be a Mom, a Web-Based Intervention to Prevent Postpartum Depression: The Enhancement of Self-Regulatory Skills and Its Association With Postpartum Depressive Symptoms | [58] | Low |  | High | Low | Low | Low |
| Fonseca et al. (2020) | Be a Mom, a Web-Based Intervention to Prevent Postpartum Depression: Results From a Pilot Randomized Controlled Trial | [57] | Some concerns |  | Some concerns | Low | Some concerns | Some concerns |
| Forsell et al. (2017) | Internet delivered cognitive behavior therapy for antenatal depression: A randomised controlled trial | [20] | Low |  | Low | Low | Low | Low |
| Gammer et al. (2020) | A Randomized Controlled Trial of an Online, Compassion-Based Intervention for Maternal Psychological Well-Being in the First Year Postpartum | [59] | Low |  | Low | Low | Low | Low |
| Gjerdingen et al. (2013) | Postpartum Doula and Peer Telephone Support for Postpartum Depression: A Pilot Randomized Controlled Trial | [49] | Some concerns |  | High | Low | Low | High |
| Guo et al. (2020) | Preventing Postpartum Depression With Mindful Self-Compassion Intervention - A Randomized Control Study | [65] | Low |  | High | Low | Low | High |
| Haga et al. (2018) | Mamma Mia – A randomized controlled trial of an internet-based intervention for perinatal depression | [37] | Low |  | High | Low | Low | High |
| Haga et al. (2020) | Mamma Mia – A randomized controlled trial of an internet intervention to enhance subjective well-being in perinatal women | [74] | Low |  | Low | Low | Low | Low |
| Heller et al. (2020) | The Effectiveness of a Guided Internet-Based Tool for the Treatment of Depression and Anxiety in Pregnancy (MamaKits Online): Randomized Controlled Trial | [38] | Low |  | High | High | Low | High |
| Ishola & Chipps (2015) | The use of mobile phones to deliver acceptance and commitment therapy in the prevention of mother–child HIV transmission in Nigeria | [73] | Low |  | Low | Low | Low | Low |
| Jiao et al. (2019) | Web-based versus home-based postnatal psychoeducational interventions for first-time mothers: A randomised controlled trial | [75] | Low |  | Low | Low | Low | Low |
| Kalmbach et al. (2020) | A randomized controlled trial of digital cognitive behavioral therapy for insomnia in pregnant women | [50] | Low |  | Low | Low | Low | Low |
| Kelman et al. (2017) | A proof‐of‐concept pilot randomized comparative trial of brief Internet‐based compassionate mind training and cognitivebehavioral therapy for perinatal and intending to become pregnant women | [76] | Low |  | High | Low | Low | High |
| Krusche et al. (2018) | Mindfulness for pregnancy: A randomised controlled study of online mindfulness during pregnancy | [60] | Some concerns |  | High | Low | Low | High |
| Loughnan et al. (2019) | A randomized controlled trial of ‘MUMentum Pregnancy’: Internet-delivered cognitive behavioral therapy program for antenatal anxiety and depression | [39] | Low |  | High | Low | Low | High |
| Loughnan et al. (2019) | A randomised controlled trial of ‘MUMentum postnatal’: Internet-delivered cognitive behavioural therapy for anxiety and depression in postpartum women | [70] | Low |  | Low | Low | Low | Low |
| Milgrom et al. (2016) | Internet Cognitive Behavioral Therapy for Women With Postnatal Depression: A Randomized Controlled Trial of MumMoodBooster | [71] | Low |  | Low | Low | Low | Low |
| Monteiro et al. (2020) | Be a Mom’s Ecacy in Enhancing Positive Mental Health among PostpartumWomen Presenting Low Risk for Postpartum Depression: Results from a Pilot Randomized Trial | [61] | Low |  | Some concerns | Low | Low | Some concerns |
| Ngai et al. (2015) | The Effect of Telephone-Based Cognitive-Behavioral Therapy on Postnatal Depression: A Randomized Controlled Trial | [66] | Low |  | Low | Low | Low | Low |
| Nieminen et al. (2016) | Internet-provided cognitive behaviour therapy of posttraumatic stress symptoms following childbirth—a randomized controlled trial | [62] | Low |  | High | Low | Low | Low |
| O'Mahen et al. (2013) | Internet-based behavioral activation—Treatment for postnatal depression (Netmums): A randomized controlled trial | [63] | Low |  | High | Low | Low | High |
| Posmontier et al. (2016) | Telephone-Administered Interpersonal Psychotherapy by Nurse-Midwives for Postpartum Depression | [51] | High |  | Some concerns | Low | Low | High |
| Pugh et al. (2016) | A Randomised Controlled Trial of Therapist-Assisted, Internet-Delivered Cognitive Behavior Therapy for Women with Maternal Depression | [52] | Low |  | Low | Low | Low | Low |
| Sawyer et al. (2019) | The Effectiveness of an App-Based Nurse-Moderated Program for New Mothers With Depression and Parenting Problems (eMums Plus): Pragmatic Randomized Controlled Trial | [72] | Low |  | Low | Low | Low | Low |
| Scherer et al. (2016) | Patient satisfaction and psychological well-being after internet-based cognitive behavioral stress management (IB-CBSM) for women with preterm labor: A randomized controlled trial | [64] | Low |  | Some concerns | Low | Low | Some concerns |
| Sheeber et al. (2012) | Development and Pilot Evaluation of an Internet-Facilitated Cognitive-Behavioral Intervention for Maternal Depression | [41] | Low |  | Low | Low | Low | Low |
| Shorey et al. (2017) | A randomized-controlled trial to examine the effectiveness of the ‘Home-but not Alone’ mobile-health application educational programme on parental outcomes | [68] | Low |  | High | Low | Low | Low |
| Shorey et al. (2019) | Evaluation of a Technology-Based Peer-Support Intervention Program for Preventing Postnatal Depression (Part 1): Randomized Controlled Trial | [67] | Low |  | Low | Low | Low | Low |
| Shorey et al. (2019) | Effectiveness of a Technology-Based Supportive Educational Parenting Program on Parental Outcomes (Part 1): Randomized Controlled Trial | [77] | Low |  | Low | Low | Low | Low |
| Stremler et al. (2013) | Effect of behavioural-educational intervention on sleep for primiparous women and their infants in early postpartum: multisite randomised controlled trial | [53] | Low |  | Low | Low | Low | Low |
| Urech et al. (2017) | Efficacy of an internet-based cognitive behavioral stress management training in women with idiopathic preterm labor: A randomized controlled intervention study | [40] | Low |  | Low | Low | Low | Low |
| Wozney et al. (2017) | Strongest Families™ Managing Our Mood (MOM): a randomized controlled trial of a distance intervention for women with postpartum depression | [54] | Low |  | High | Low | Low | Low |
| Yang et al. (2019) | Optional Web-Based Videoconferencing Added to Office-Based Care for Women Receiving Psychotherapy During the Postpartum Period: Pilot Randomized Controlled Trial | [69] | Low |  | Low | Low | Low | Low |
| Yang et al. (2019) | Effects of an Online Mindfulness Intervention Focusing on Attention Monitoring and Acceptance in Pregnant Women: A Randomized Controlled Trial | [55] | Low |  | Low | Some concerns | Low | Some concerns |
| *D2: skipped, because blinding is usually not possible due to the nature of the intervention | | | | | | | | |
