# Supplementary material for: Mental health-related telemedicine interventions for pregnant women and new mothers: a systematic literature review
Source: BMC Psychiatry. 2023 Apr 28;23:292. doi: 10.1186/s12888-023-04790-0 (PMC10148488; doi:10.1186/s12888-023-04790-0)
Supplement: Supplementary file 6 [file 12888_2023_4790_MOESM1_ESM.docx]

Telemedical interventions for mentally stressed, pregnant women and young mothers: protocol for a systematic literature review

Ulrike Stentzel, Angelika Beyer, Neeltje van den Berg

Correspondence: ulrike.stentzel@uni-greifswald.de

Keywords: telemedicine, telemedical interventions, mobile Health, mentally stressed, mental disorder, pregnancy, young mothers

# Abstract

Pregnancy and puerperium are times with a higher risk for depression and mental strain in women. An untreated mental disorder during pregnancy, especially a depression or anxiety, can affect the mother but also the fetus as well as the child after given birth. Treatment rates are low. Thus, interventions with a low-threshold-access for pregnant women and young mothers are needed. Telemedical interventions are promising. The objective of this study is to obtain the current scientific evidence to the effects of telemedical interventions for mentally stressed pregnant women and young mothers and the effects of telemedical interventions on the women’s prepartum and postpartum mental health/ mental strain/ mental stress. This review will consider all studies that involve pregnant women or young mothers that are affected by mental stress or some kind of a mental disorder or disease. All trials with an established study designs will be considered. The trials need to address an explicit endpoint that should affect mental conditions. The administered interventions have to be telemedical interventions of any kind. Several comparisons will be relevant to include like telemedical intervention with and without personal contacts. All kinds of outcomes will be included and any type of setting. 

# Background

Pregnancy and puerperium are times with a higher risk for depression and mental strain in women. Because hormonal changes increase the risk for mental disorders, the vulnerability for affective disorders is higher during the childbearing years (1). In high-income countries, 10 % of pregnant women and 13 % of young mothers suffer from some type of mental disorders (2). In Germany, the analysis of reimbursement data of a German statutory health insurance showed a prevalence of 9.3 % for depression, 16.9 % for anxiety disorder, 24.2 % for a somatoform/dissociative disorder, and 11.7 % for acute stress reactions in pregnant women. In total, 43.6 % of 38,174 pregnant women had at least one disease in the field of mental disorders in 2008 (3). Within the course of the pregnancy, the risk for depression is higher in the second (12.8 %) and third trimester (12.0 %) compared to the the first (7.4 %) (4). An untreated mental disorder during pregnancy, especially a depression or anxiety, can affect the mother but also the fetus as well as the child after given birth (5). The mother is often affected by poor nutrition and impaired self-care, a failure to follow medical and prenatal guidelines, a worsening of comorbid medical illness, an increased exposure to tobacco, alcohol and drugs, postpartum occurring psychiatric complications and by impacts on family members. The fetus is affected by preterm birth, lower birth weight, reduced fetal growth, a higher risk for spontaneous abortion, a higher risk for pre-eclampsia and by a higher risk for operative or instrumental delivery. The newborns have a higher risk for a poor neonatal adaptation; stays at the neonatal ICU are more likely. Apgar scores can be lower and head circumferences can be smaller. They show more often growth retardation, a slowed mental development, excessive crying, irritability, hostility and erratic sleep. Regarding the child development, untreated mental disorders in the mothers can affect the mother-child-bonding. It can cause cognitive delays, behavioral and emotional difficulties, maladaptive social interactions and difficulties with affect regulation. Affected children display more fear and anxiety, higher rates of ADHD, higher impulsivity and lower IQ at age 14 - 15 years (5). It is also known that children with mentally ill parents have higher risks to develop a psychopathology of their own (6-8). Hence, mental disorders during pregnancy and puerperium is serious for both mother and child, and diagnostic and treatment is needed (9, 10). Nevertheless, treatment rates are low (11). Flynn et al. found, that 65 % of pregnant women with current major depressive disorder were not receiving any depression treatment (12). Stigma, risks of using antidepressant medications during pregnancy, lack of time, childcare issues, as well as limited resources in rural regions, high amounts of stress, lack of transportation and inadequate support were identified as impeding barriers to care (10, 13). Thus, interventions with a low-threshold-access and care that reaches also rural and remote residing affected pregnant women and young mothers are needed. Addressing these mentioned issues telemedical approaches are promising. Telemedical interventions can replace face-to-face care but can also support and exonerate the usual care.

If amendments of this protocol may become necessary, the date of each amendment, a description of the alterations and the rationale will be given according to the AHRQ process for dealing with protocol amendments. 

1.	Gawlik S, Waldeier L, Muller M, Szabo A, Sohn C, Reck C. Subclinical depressive symptoms during pregnancy and birth outcome--a pilot study in a healthy German sample. Archives of women's mental health. 2013;16(2):93-100.

2.	Fisher J, Cabral de Mello M, Patel V, Rahman A, Tran T, Holton S, et al. Prevalence and determinants of common perinatal mental disorders in women in low- and lower-middle-income countries: a systematic review. Bulletin of the World Health Organization. 2012;90(2):139g-49g.

3.	Wallwiener S, Goetz M, Lanfer A, Gillessen A, Suling M, Feisst M, et al. Epidemiology of mental disorders during pregnancy and link to birth outcome: a large-scale retrospective observational database study including 38,000 pregnancies. Archives of gynecology and obstetrics. 2019;299(3):755-63.

4.	Bennett HA, Einarson A, Taddio A, Koren G, Einarson TR. Prevalence of depression during pregnancy: systematic review. Obstetrics and gynecology. 2004;103(4):698-709.

5.	Fishell A. Depression and anxiety in pregnancy. Journal of population therapeutics and clinical pharmacology = Journal de la therapeutique des populations et de la pharamcologie clinique. 2010;17(3):e363-9.

6.	Beardslee WR, Versage EM, Gladstone TR. Children of affectively ill parents: a review of the past 10 years. Journal of the American Academy of Child and Adolescent Psychiatry. 1998;37(11):1134-41.

7.	Verrocchio MC, Ambrosini A, Fulcheri M. Promotion of mental health in children of parents with a mental disorder. Annali dell'Istituto superiore di sanita. 2013;49(4):406-11.

8.	Apter G, Bobin A, Genet MC, Gratier M, Devouche E. Update on Mental Health of Infants and Children of Parents Affected With Mental Health Issues. Current psychiatry reports. 2017;19(10):72.

9.	O'Connor E, Rossom RC, Henninger M, Groom HC, Burda BU. Primary Care Screening for and Treatment of Depression in Pregnant and Postpartum Women: Evidence Report and Systematic Review for the US Preventive Services Task Force. Jama. 2016;315(4):388-406.

10.	Forsell E, Bendix M, Hollandare F, Szymanska von Schultz B, Nasiell J, Blomdahl-Wetterholm M, et al. Internet delivered cognitive behavior therapy for antenatal depression: A randomised controlled trial. Journal of affective disorders. 2017;221:56-64.

11.	Baker-Ericzen MJ, Connelly CD, Hazen AL, Duenas C, Landsverk JA, Horwitz SM. A collaborative care telemedicine intervention to overcome treatment barriers for Latina women with depression during the perinatal period. Families, systems & health : the journal of collaborative family healthcare. 2012;30(3):224-40.

12.	Flynn HA, O'Mahen HA, Massey L, Marcus S. The impact of a brief obstetrics clinic-based intervention on treatment use for perinatal depression. Journal of women's health (2002). 2006;15(10):1195-204.

13.	Evans EC, Deutsch NL, Drake E, Bullock L. Nurse-Patient Interaction as a Treatment for Antepartum Depression: A Mixed-Methods Analysis [Formula: see text]. Journal of the American Psychiatric Nurses Association. 2017;23(5):347-59.

# Objectives

## Objectives (Initial):

The objective of this study is to obtain the current scientific evidence to the effects of telemedical interventions for mentally stressed pregnant women and young mothers and the effects of telemedical interventions on the women’s prepartum and postpartum mental health/ mental strain/ mental stress. The specific review questions to be addressed are:

- For which kind of mental stress, mental disorders, mental diseases or mental diagnoses in pregnant women and young mothers telemedical interventions and concepts have been developed and evaluated?

- Which kind of telemedical interventions (applications, modalities, targets) were developed and conducted for the specified patient group? 

- Were the telemedical interventions feasible to reduce mental stress in pregnant women and in young mothers? (Telemedical interventions on its own or in addition to usual care?)

- What kind of telemedical interventions showed promising results in reducing mental stress/mental conditions? Which characteristics were successful, such like in technical terms (intervention provided via phone calls, apps, web portals)? Where there differences between interventions using only a technical device and interventions with (additional) personal support? Where there differences in the success regarding the delivery timeframe per- versus postpartum?

- How was the acceptance by the participants?

- Were implementation barriers reported and if so, what kind of?

Which kind of outcomes can be addressed with telemedical interventions?

## PICO Schema (initial)

| Primary/secondary | Question type | Question | Definition key elements |
| --- | --- | --- | --- |
| primary | P I/E C O | For which kind of mental stress, mental disorders, mental diseases or mental diagnoses in pregnant women and young mothers telemedical interventions and concepts have been developed and evaluated? | Population: pregnant women and young mothers Intervention/Exposure: telemedical interventions and concepts Comparator: telemedical intervention versus non-telemedical intervention, with personal contact or feedback versus purely technical telemedical intervention, delivered prepartum versus postpartum Outcome: mental stress, mental disorders, mental diseases or mental diagnoses |
| secondary | P I/E C O | Which kind of telemedical interventions (applications, modalities, targets) were developed and conducted for the specified patient group? | Population: pregnant women and young mothers  Intervention/Exposure: telemedical interventions and concepts  Comparator:  Outcome: mental stress, mental disorders, mental diseases or mental diagnoses |
| secondary | P O | Were the telemedical interventions feasible to reduce mental stress in pregnant women and in young mothers? (Telemedical interventions on its own or in addition to usual care?) | Population: pregnant women and in young mothers Outcome: reduce mental stress |
| secondary | P O | What kind of telemedical interventions showed promising results in reducing mental stress/mental conditions? Which characteristics were successful, such like in technical terms (intervention provided via phone calls, apps, web portals)? Where there differences between interventions using only a technical device and interventions with (additional) personal support? Where there differences in the success regarding the delivery timeframe per- versus postpartum? | Population: pregnant women and young mothers Outcome: kind of telemedical interventions, telemedical application characteristics, |
| secondary |  | How was the acceptance by the participants? |
| secondary |  | Were implementation barriers reported and if so, what kind of? |
| secondary |  | Which kind of outcomes can be addressed with telemedical interventions? |

## Amendment Objectives (04.12.2020)

During the screening process it became obvious that the research question lacked structure and clarity. Hence, the objective was revised giving the research question more structure through allocation to primary and secondary priority and more focus through more precise wording. The final questions are the following:

- Primary objective: Can telemedicine interventions reduce mental stress in pregnant women and young mothers?

- Secondary objectives:

  - Which kind of telemedical interventions (applications, modalities, targets) were developed and conducted for the specified patient group? 

  - For which kind of mental stress, mental disorders, mental diseases or mental diagnoses in pregnant women and young mothers telemedical interventions have been developed and evaluated?

  - Which characteristics were successful, such like in technical terms (intervention provided via phone calls, apps, web portals)? Where there differences between interventions using only a technical device and interventions with (additional) personal support? Where there differences in the success regarding the delivery timeframe per- versus postpartum?

  - How was the acceptance by the participants?

  - Were implementation barriers reported and if so, what kind of?

  - Which kind of outcomes can be addressed with telemedical interventions?

## Amendment PICO Schema 

| Primary/secondary | Question type | Question | Definition key elements |
| --- | --- | --- | --- |
| primary | P I/E C O | Can telemedicine interventions reduce mental stress in pregnant women and young mothers? | Population: pregnant women and young mothers Intervention/Exposure: telemedical interventions Comparator: telemedical intervention versus TAU, other (non-telemedical) interventions Outcome: mental stress, mental disorders, mental diseases or mental diagnoses |
| secondary | P I/E C O | Which kind of telemedical interventions (applications, modalities, targets) were developed and conducted for the specified patient group? | Population: pregnant women and young mothers  Intervention/Exposure: telemedical interventions Comparator:  Outcome: mental stress, mental disorders, mental diseases or mental diagnoses |
| secondary | P I/E C O | For which kind of mental stress, mental disorders, mental diseases or mental diagnoses in pregnant women and young mothers telemedical interventions have been developed and evaluated? | Population: pregnant women and young mothers  Intervention/Exposure: telemedical interventions Comparator:  Outcome: mental stress, mental disorders, mental diseases or mental diagnoses |
| secondary | P O | Which characteristics were successful, such like in technical terms (intervention provided via phone calls, apps, web portals)? | Population: pregnant women and young mothers Outcome: kind of telemedical interventions, telemedical application characteristics, |
| secondary |  | How was the acceptance by the participants? |
| secondary |  | Were implementation barriers reported and if so, what kind of? |
| secondary |  | Which kind of outcomes can be addressed with telemedical interventions? |

# Search Strategy

## Information sources and search strategy (Initial)

The literature search will be conducted within the following databases:

- Pubmed, Medline, PMC

- Cochrane

- Isi Web of Science

- Science Direct

- Google Scholar (for grey literature)

The search term contains the terms “telemedicine”, “mental stress”, “pregnant women” and “young mothers” and numerous synonyms for these terms (see Table 1), including medical subject headings (MeSH).

Table 1: Search terms

| Concept | Search terms |
| --- | --- |
| telemedicine | telemedicine, telemedical, tele care, Mobile Health, telehealth, telepsychiatry, eHealth, mhealth, phone call, text messages, smartphone, tablet, apps, web apps, web portal, internet |
| mental stress | mentally stressed, mental health, mental disorder, mental disease, mentally ill, psychological, psychological impairment, psychologically affected, psychologically stressed, psychological disorder, psychological distress, psychiatric disorder, psychiatric disease, depression, anxiety, panic disorder |
| pregnant women | pregnancy, pregnant, prenatal, prepartal, antenatal, prepartum, peripartum, gestation, gravidic, childbearing |
| young mothers | young mothers, new mums/moms, young mums/moms, peripartum, postnatal, postpartal, postpartum, Puerperium |

A transcript of a draft search strategy for PubMed is given in the appendix. 

## Amendment to information sources and search strategy (20.05.2020)

Initially the databases that were aimed for the literature search were:

- Pubmed, Medline, PMC

- Cochrane

- Isi Web of Science

- Science Direct

- Google Scholar (for grey literature)

Actual preparing and conducting the searches in those databases revealed, that not every of the above named databases were qualified for advanced searches using substantial search terms, combined through operators. Hence, two of the databases were exchanged against one other. Unfortunately, this also applies to the database, which should be used for grey literature. The authors will use the following databases:

- Pubmed, Medline, PMC

- Cochrane

- Isi Web of Science

- PsychInfo.

# Study inclusion criteria

## Eligibility criteria (Initial)

This review will consider all studies that involve pregnant women or young mothers that are affected by mental stress or some kind of a mental disorder or disease basing on an established study design. The trials need to address an explicit endpoint that should affect mental conditions. The administered interventions have to be telemedical interventions of any kind. In addition, feasibility and implementation studies of telemedical interventions in the field of mental disorders in pregnant women and young mothers will be of interest too.

Interventions that fulfill the inclusion criteria are all reported interventions with a telemedical approach, involving the above described participants and conditions. The interventions have in common that they are rendered over spatial distances via information and communication technologies. The intervention has to address the mental condition of the participants in some form of personal intervention e.g. telephone calls, chats, videoconferences or interactive support system e.g. apps for smartphone or tablet or web portals. 

Given the broad perspective for interventions of interest, several comparisons will be relevant to include:

- Health condition with telemedical intervention versus non-telemedical intervention.

- Health condition with telemedical intervention that includes also personal contact or feedback versus purely technical telemedical intervention.

- Feasibility of telemedical interventions delivered prepartum versus delivered postpartum.

All kinds of outcomes (e.g. medical outcomes, patient oriented outcomes) will be included. There will be no restrictions by type of setting.

Articles in English and German language will be considered. Abstracts must be available. Because of technical developments of telemedical systems and devices as well as the increase of the number of households with access to the internet, the review was restricted to studies as from 2007 until August 2019. This restriction of the time period reduces heterogeneity due to technical development and communication infrastructure thus improving comparability between the studies.

Commentaries, letters, editorials, cross-sectional studies, case series, and case reports will not be included. 

## Amendment Eligibility criteria (04.12.2020)

Because of time progress the search was newly done with the search time frame of 2007 until November 15, 2020. Initially, all study types should be included in the review, as it was unclear how many suitable studies could be found. Then it became apparent that due to the number of studies found, the review could be limited to the most significant study types, the RCT. Therefore, the eligibility criteria were constrained to the study type RCT. The final text will be the following:

This review will consider randomized controlled trials (RCT) that involve pregnant women or young mothers that are affected by mental stress or some kind of a mental disorder or disease basing on an established study design. The trials need to address an explicit endpoint that should affect mental conditions. 

Interventions that fulfill the inclusion criteria are all reported interventions with a telemedical approach, involving the above described participants and conditions. The interventions have in common that they are rendered over spatial distances via information and communication technologies. The intervention has to address the mental condition of the participants in some form of personal intervention e.g. telephone calls, chats, videoconferences or interactive support system e.g. apps for smartphone or tablet or web portals. 

Given the broad perspective for interventions of interest, several comparisons will be relevant to include:

- Health condition with telemedical intervention versus non-telemedical intervention.

- Health condition with telemedical intervention that includes also personal contact or feedback versus purely technical telemedical intervention.

- Feasibility of telemedical interventions delivered prepartum versus delivered postpartum.

All kinds of outcomes (e.g. medical outcomes, patient oriented outcomes) will be included. There will be no restrictions by type of setting.

Articles in English and German language will be considered. Abstracts must be available. Because of technical developments of telemedical systems and devices as well as the increase of the number of households with access to the internet, the review was restricted to studies as from 2007 until November 16, 2020. This restriction of the time period reduces heterogeneity due to technical development and communication infrastructure thus improving comparability between the studies.

# Study screening mode

## Study records

The review will be conducted on the basis of CADIMA, an open access online tool that supports the conduction und the reporting of systematic reviews and systematic maps [14]. CADIMA facilitates the 

- set up of the review (including to define the research question) 

- protocol development (including inclusion and exclusion criteria), 

- duplicate checking, 

- article screening/study selection (also for a team of reviewers)

- and critical appraisal/risk of bias assessments. 

The literature search results will be uploaded in CADIMA. The full text of articles that met the inclusion criteria by title and abstract or inconclusive articles will be obtained by US. The screening for the inclusion criteria will be conducted by two reviewers (US and AB) independently within CADIMA. Differences in the screening results will be discussed and solved in researcher team meetings. CADIMA provides also functionalities for data extraction, result presentation or for critical appraisal (risk of bias assessments). To assess the possible risk of bias, the Cochrane Collaboration tool for assessing the risk of bias [15] (Table 8.5.a in the Cochrane Handbook for Systematic Reviews of Interventions http://handbook-5-1.cochrane.org/) will also be used. Within this tool generation of random allocation sequence; concealment of the allocation sequence; blinding of participants, healthcare providers, data collectors, and outcome adjudicators; and proportion of patients lost to follow-up will be assessed. 

## Critical appraisal of included studies 

To assess the possible risk of bias, the Cochrane Collaboration tool for assessing the risk of bias (1) will be used. Within this tool generation of random allocation sequence; concealment of the allocation sequence; blinding of participants, healthcare providers, data collectors, and outcome adjudicators; and proportion of patients lost to follow-up will be assessed. The tool is described in the Cochrane Handbook for Systematic Reviews of Interventions (2). Instructions, examples and tables are provided on [http://handbook-5-1.cochrane.org/](http://handbook-5-1.cochrane.org/). Especially the tables 8.5.a and 8.5.d will serve as templates for the proposed review. Table 8.5.a contains a list of bias domains and their support for judgement and review authors’ judgement. The listed bias domains are selection bias (subdivided into random sequence generation and allocation concealment), performance bias, detection bias, attrition bias, reporting bias and other bias. The biases will be judged as ‘Low risk’, ‘High risk’ or ‘Unclear risk’ of bias. Table 8.5.d provides the specific criteria for judging the risk of different bias types. 

1.	Higgins JPT, Altman DG, Gøtzsche PC, Jüni P, Moher D, Oxman AD, et al. The Cochrane Collaboration's tool for assessing risk of bias in randomised trials. BMJ (Clinical research ed). 2011;343:d5928-d.

2.	Higgins JP, Green S. Cochrane handbook for systematic reviews of interventions: John Wiley & Sons; 2011.

## Amendment Critical appraisal (05.02.2021)

Between the preparation of the protocol submitted to PROSPERO and the actual execution of the Risk of Bias assessment, the Risk of Bias tool was revised and a new version published. Now, to assess the possible risk of bias, the revised Cochrane risk-of-bias tool for randomized trials (RoB 2).

## Critical appraisal mode

The critical appraisal will be done by two authors and will be performed within CADIMA. In CADIMA in the section of the data extraction the critical appraisal criteria were defined according to the Cochrane Collaboration tool for assessing the risk of bias by following the instructions of the Cochrane Handbook for Systematic Reviews of Interventions. 

# Data extraction strategy

For each included article, the following information shall be systematically extracted and presented in a results table: Author details (author names, publication title, date of publication), description of participants, study type and design description, study setting and country, number of included participants, number of drop outs, participants’ medical condition/type of diagnoses, description of the intervention (including benefits and harms), outcome and main results, risk of bias. The extraction process will be conducted with a structured data extraction worksheet, supported by the extraction functionalities of CADIMA. CADIMA also supports duplicate checking. Afterwards, the results will be compared and differences or similarities between the studies will be highlighted. 

# Data analysis

The quantitative results of the included studies will be presented in tables and/or charts. A systematic narrative synthesis (1, 2) will be performed with information presented in the text and tables to summarize and explain the characteristics and findings of the included studies. The synthesis will be divided into RCTs and feasibility studies. For each included article, the following information shall be systematically extracted and presented in a results table: Author details (author names, publication title, date of publication), description of participants, study type and design description, study setting and country, number of included participants, number of drop outs, participants’ medical condition/type of diagnoses, description of the intervention (including benefits and harms), outcome and main results, risk of bias. After that, the results will be compared and differences or similarities between the studies will be highlighted. The above detailed mentioned research questions as well as comparators will serve as a framework for the narrative synthesis.

This review aims on gaining an overall overview about telemedical interventions that were designed, implemented and evaluated for a very specific patient group. The patient group is the key element. Telemedical interventions that focusses on pregnant women and young mothers and addresses some kind of mental stress, mental disorders, mental diseases or mental diagnoses shall be compiled. Hence, the range and approaches of reported telemedical interventions for this group will be broad and various. As such, a description of the strength of the body of evidence is not applicable for this review.

1.	Prictor M, Hill S. Cochrane Consumers and Communication Review Group: leading the field on health communication evidence. Journal of evidence-based medicine. 2013;6(4):216-20.

2.	Ryan R. Cochrane Consumers and Communication Review Group: data synthesis and analysis. Cochrane Consumers and Communication Review Group; 2013.

# Competing interests and sources of support 

The author(s) declare that they have no competing interests. 

This systematic review will be conducted within the scope of the German research project “Psychological and neurobiological health in pregnant women and young mothers (PriVileG-M)“ that is funded by the Landesamt für Gesundheit und Soziales Mecklenburg-Vorpommern (Federal Office for Health and Social Affairs Mecklenburg-Western Pomerania). The funding occurs on the basis of the operational program 2014-2020 for Mecklenburg-Western Pomerania using funds originating from the European Social Fund (ESF). The funding agency is neither involved in the content and the design of the study or the compilation, analysis and interpretation of the data nor in the drafting of this review protocol and the review itself or in the decision to submit the results for publication. The funding has the purpose to promote young scientists. The funding is approved according to the Directive for the Promotion of Young Scientists in Excellence research alliances. 

# Authors' contributions

US drafted the manuscript. US and NvdB contributed to the development of the selection criteria, the risk of bias assessment strategy and data extraction criteria. US and AB will participate in the identification of the relevant articles and will assess the risk of bias. NvdB will proof read the manuscript, will provide feedback and will finally approve the manuscript.  

# Appendencies

## Search term for pregnant women

### Initial search term

((((((((((((((((telemedicine[Title/Abstract]) OR web portal[Title/Abstract]) OR web apps[Title/Abstract]) OR apps[Title/Abstract]) OR tablet[Title/Abstract]) OR smartphone[Title/Abstract]) OR text messages[Title/Abstract]) OR phone call[Title/Abstract]) OR mhealth[Title/Abstract]) OR eHealth[Title/Abstract]) OR telepsychiatry[Title/Abstract]) OR telehealth[Title/Abstract]) OR Mobile Health[Title/Abstract]) OR tele care[Title/Abstract]) OR telemedical[Title/Abstract]) OR internet[Title/Abstract]) AND ((((((((((((((mentally stressed[Title/Abstract]) OR mental health[Title/Abstract]) OR mental disorder[Title/Abstract]) OR mental disease[Title/Abstract]) OR mentally ill[Title/Abstract]) OR psychological[Title/Abstract]) OR psychological impairment[Title/Abstract]) OR psychologically affected[Title/Abstract]) OR psychologically stressed[Title/Abstract]) OR psychological disorder[Title/Abstract]) OR psychological distress[Title/Abstract]) OR psychiatric disorder[Title/Abstract]) OR psychiatric disease[Title/Abstract]) OR depression[Title/Abstract]) AND ((((((((((pregnancy[Title/Abstract]) OR pregnant[Title/Abstract]) OR prenatal[Title/Abstract]) OR prepartal[Title/Abstract]) OR antenatal[Title/Abstract]) OR prepartum[Title/Abstract]) OR peripartum[Title/Abstract]) OR gestation[Title/Abstract]) OR gravidic[Title/Abstract]) OR childbearing[Title/Abstract])

### Amendment Search term for pregnant women (16.11.2020)

The search term was slightly amended.

### Final search term

Search: (((((((((((((((((telemedicine[Title/Abstract]) OR "tele psychiatry" [Title/Abstract]) OR web [Title/Abstract]) OR apps[Title/Abstract]) OR applications[Title/Abstract]) OR tablet[Title/Abstract]) OR smartphone[Title/Abstract]) OR "text messages"[Title/Abstract]) OR "phone call"[Title/Abstract]) OR mhealth[Title/Abstract]) OR eHealth[Title/Abstract]) OR telepsychiatry[Title/Abstract]) OR telehealth[Title/Abstract]) OR "Mobile Health"[Title/Abstract]) OR "tele care"[Title/Abstract]) OR telemedical[Title/Abstract]) OR internet[Title/Abstract]) AND (((((((((((((("mentally stressed"[Title/Abstract]) OR "mental health"[Title/Abstract]) OR "mental disorder"[Title/Abstract]) OR "mental disease"[Title/Abstract]) OR "mentally ill"[Title/Abstract]) OR psychological[Title/Abstract]) OR "psychological impairment"[Title/Abstract]) OR "psychologically affected"[Title/Abstract]) OR "psychologically stressed"[Title/Abstract]) OR "psychological disorder"[Title/Abstract]) OR "psychological distress"[Title/Abstract]) OR "psychiatric disorder"[Title/Abstract]) OR "psychiatric disease"[Title/Abstract]) OR depression[Title/Abstract]) AND ((((((((((pregnancy[Title/Abstract]) OR pregnant[Title/Abstract]) OR prenatal[Title/Abstract]) OR prepartal[Title/Abstract]) OR antenatal[Title/Abstract]) OR prepartum[Title/Abstract]) OR peripartum[Title/Abstract]) OR gestation[Title/Abstract]) OR gravidic[Title/Abstract]) OR childbearing[Title/Abstract]) Filters: from 2007/1/1 - 2019/12/31

## Search term for young mothers

### Initial search term

((((((((((((((((telemedicine[Title/Abstract]) OR web portal[Title/Abstract]) OR web apps[Title/Abstract]) OR apps[Title/Abstract]) OR tablet[Title/Abstract]) OR smartphone[Title/Abstract]) OR text messages[Title/Abstract]) OR phone call[Title/Abstract]) OR mhealth[Title/Abstract]) OR eHealth[Title/Abstract]) OR telepsychiatry[Title/Abstract]) OR telehealth[Title/Abstract]) OR Mobile Health[Title/Abstract]) OR tele care[Title/Abstract]) OR telemedical[Title/Abstract]) OR internet[Title/Abstract]) AND (((((((((((((mentally stressed[Title/Abstract]) OR mental health[Title/Abstract]) OR mental disorder[Title/Abstract]) OR mental disease[Title/Abstract]) OR mentally ill[Title/Abstract]) OR psychological[Title/Abstract]) OR psychological impairment[Title/Abstract]) OR psychologically affected[Title/Abstract]) OR psychologically stressed[Title/Abstract]) OR psychological disorder[Title/Abstract]) OR psychological distress[Title/Abstract]) OR psychiatric disorder[Title/Abstract]) OR psychiatric disease[Title/Abstract]) AND ((((((((((young mothers[Title/Abstract]) OR new mums[Title/Abstract]) OR new moms[Title/Abstract]) OR young mums[Title/Abstract]) OR young moms[Title/Abstract]) OR peripartum[Title/Abstract]) OR postnatal[Title/Abstract]) OR postpartal[Title/Abstract]) OR postpartum[Title/Abstract]) OR Puerperium[Title/Abstract])

### Amendment Search term for young mothers (16.11.2020)

The search term was slightly amended.

### Final search term

Search: (((((((((((((((((telemedicine[Title/Abstract]) OR "tele psychiatry" [Title/Abstract]) OR web [Title/Abstract]) OR apps[Title/Abstract]) OR applications[Title/Abstract]) OR tablet[Title/Abstract]) OR smartphone[Title/Abstract]) OR "text messages"[Title/Abstract]) OR "phone call"[Title/Abstract]) OR mhealth[Title/Abstract]) OR eHealth[Title/Abstract]) OR telepsychiatry[Title/Abstract]) OR telehealth[Title/Abstract]) OR "Mobile Health"[Title/Abstract]) OR "tele care"[Title/Abstract]) OR telemedical[Title/Abstract]) OR internet[Title/Abstract]) AND (((((((((((((("mentally stressed"[Title/Abstract]) OR "mental health"[Title/Abstract]) OR "mental disorder"[Title/Abstract]) OR "mental disease"[Title/Abstract]) OR "mentally ill"[Title/Abstract]) OR psychological[Title/Abstract]) OR "psychological impairment"[Title/Abstract]) OR "psychologically affected"[Title/Abstract]) OR "psychologically stressed"[Title/Abstract]) OR "psychological disorder"[Title/Abstract]) OR "psychological distress"[Title/Abstract]) OR "psychiatric disorder"[Title/Abstract]) OR "psychiatric disease"[Title/Abstract]) OR depression[Title/Abstract])  AND (((((((((("young mothers"[Title/Abstract]) OR "new mums"[Title/Abstract]) OR "new moms"[Title/Abstract]) OR "young mums"[Title/Abstract]) OR "young moms"[Title/Abstract]) OR peripartum[Title/Abstract]) OR postnatal[Title/Abstract]) OR postpartal[Title/Abstract]) OR postpartum[Title/Abstract]) OR Puerperium[Title/Abstract]) Filters: from 2007/1/1 - 2019/12/31
